# Guest‐Induced Reversible Phase Conversion via Spin Frustration Relief in Spin‐Intercalated Layered Antiferromagnets

**DOI:** 10.1002/advs.202507957

**Published:** 2025-07-26

**Authors:** Qingxin Liu, Honoka Nemoto, Wataru Kosaka, Hitoshi Miyasaka

**Affiliations:** ^1^ Institute for Materials Research Tohoku University 2‐1‐1 Katahira, Aoba‐ku Sendai 980–8577 Japan; ^2^ Department of Chemistry Graduate School of Science Tohoku University 6‐3 Arama‐ki‐Aza‐Aoba, Aoba‐ku Sendai 980–8578 Japan

**Keywords:** charge transfer, guest sorption, guest‐induced magnetic switching, low‐dimensional magnet, metal‐organic framework magnet, spin frustration

## Abstract

A spin‐intercalated layered metal‐organic framework (MOF) magnetic system, [MCp*_2_][{Ru_2_(2,3,5‐F_3_ArCO_2_)_4_}_2_(TCNQ)]·solv (M = Co, Fe; [MCp*_2_]^+^ = decamethylmetallocenium; 2,3,5‐F_3_ArCO_2_^−^ = 2,3,5‐trifluorobenzoate; solv = crystallization solvent) is reported, which enables reversible magnetic phase switching by controlling spin frustration. In this system, paramagnetic spins ([FeCp*_2_]^+^ with *S* = 1/2) are intercalated into a strongly correlated layered antiferromagnet, leading to competition between the interlayer antiferromagnetic coupling (*J*_LL_ < 0) and another coupling between the host and intercalated spins (*J*_LS_). The balance between these interactions governs the emergence and nature of spin frustration. When |*J*_LS_| ≤ |*J*_LL_|, the spin frustration is “evident,” resulting in a magnetic order accompanied by spin reorientation, whereas when |*J*_LS_| >> |*J*_LL_|, the frustration becomes “hidden,” and the system exhibits apparent ferromagnetic or ferrimagnetic behavior despite underlying interlayer antiferromagnetic interactions. Importantly, for the first time, a reversible transition between these two magnetic regimes is demonstrated, by controlling the solvation/desolvation of materials, which modulates the spin frustration degree without altering the intrinsic spin states. This controllable switching highlights the unique potential of spin‐intercalated molecular layered magnets as tunable platforms for studying correlated spin systems. These findings provide fundamental insights into frustration‐driven magnetic phase transitions and open new avenues for developing switchable functional materials.

## Graphical abstract

Reversible switching between “evident” (evident spin frustration, **FR‐e**) and “hidden” (hidden spin frustration, **FR‐h**) frustrated behavior is realized for the first time in spin‐intercalated layered magnets by regulating the competitive magnetic environment of *J*_LL_ and *J*_LS_ via solvent ad‐/desorption. This study underscores the utility of low‐dimensional magnetic systems in unraveling complex magnetic phenomena, with a particular focus on the interplay of competing magnetic interactions.

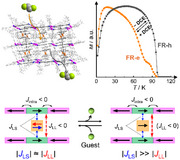

## 1 Introduction

Magnetic materials constructed from molecular building units are superior to robust inorganic magnetic materials owing to the high versatility of available building molecules and incorporable functional components, as well as their high designability in terms of structural control. We can deepen our understanding by systematically modifying each building block through molecular assembled architectures and by comparing the expected magnetic properties. Using knowledge of supramolecular and coordination chemistry, as well as Coulomb assembly with Madelung stabilization, the rational assembly of spins into multidimensional molecular frameworks and finite‐sized clusters has been achieved. Molecular magnetic materials are ideally suited for the design and construction of low‐dimensional frameworks, such as 1D chains and 2D layered structures. These low‐dimensional magnetic materials inherently enable the observation of unique physical phenomena such as the spin‐Peierls transition,^[^ ^1^, ^2^, ^3^ ^]^ Haldane gap,^[^ ^4^, ^5^, ^6^ ^]^ superparamagnetism,^[^ ^7^, ^8^, ^9^, ^10^, ^11^ ^]^ quantum spin liquids,^[^ ^12^, ^13^ ^]^ and the Berezinski‐Kosterlitz‐Thouless transition,^[^ ^14^ ^]^ rendering them ideal platforms for studying fundamental spin‐spin interaction processes. Various molecules, such as stationary molecules associated with functions of electronic spin,^[^ ^15^ ^]^ chirality,^[^ ^16^ ^]^ redox activity,^[^ ^17^, ^18^ ^]^ and conductivity,^[^ ^19^ ^]^ can be introduced into the inter‐framework void space to construct hybridized materials. Multiple and cooperative functions are expected from these hybridized materials via self‐assembly, chemical post‐modification, or alloying.^[^ ^20^, ^21^ ^]^ arious small molecules, such as volatile organic compounds (VOCs) and gas molecules, can also be introduced to generate coupling change with dynamic mass transport.^[^ ^22^, ^23^, ^24^, ^25^, ^26^, ^27^, ^28^, ^29^ ^]^ Low‐dimensional compounds have fewer constraints for crystal packing and a high degree of spatial freedom, enabling the realization or modulation of physical properties via mass transport through the pores between the frameworks.^[^ ^30^ ^]^ The flexibility of molecular materials against structural changes also contributes to the utility of pores. Therefore, molecular insertion is regarded as a perturbation with immense diversity that can modulate the physical properties of the framework without changing its components. Hence, it will be an essential tool in the future development of molecular solid‐state chemistry.

**Scheme 1:**
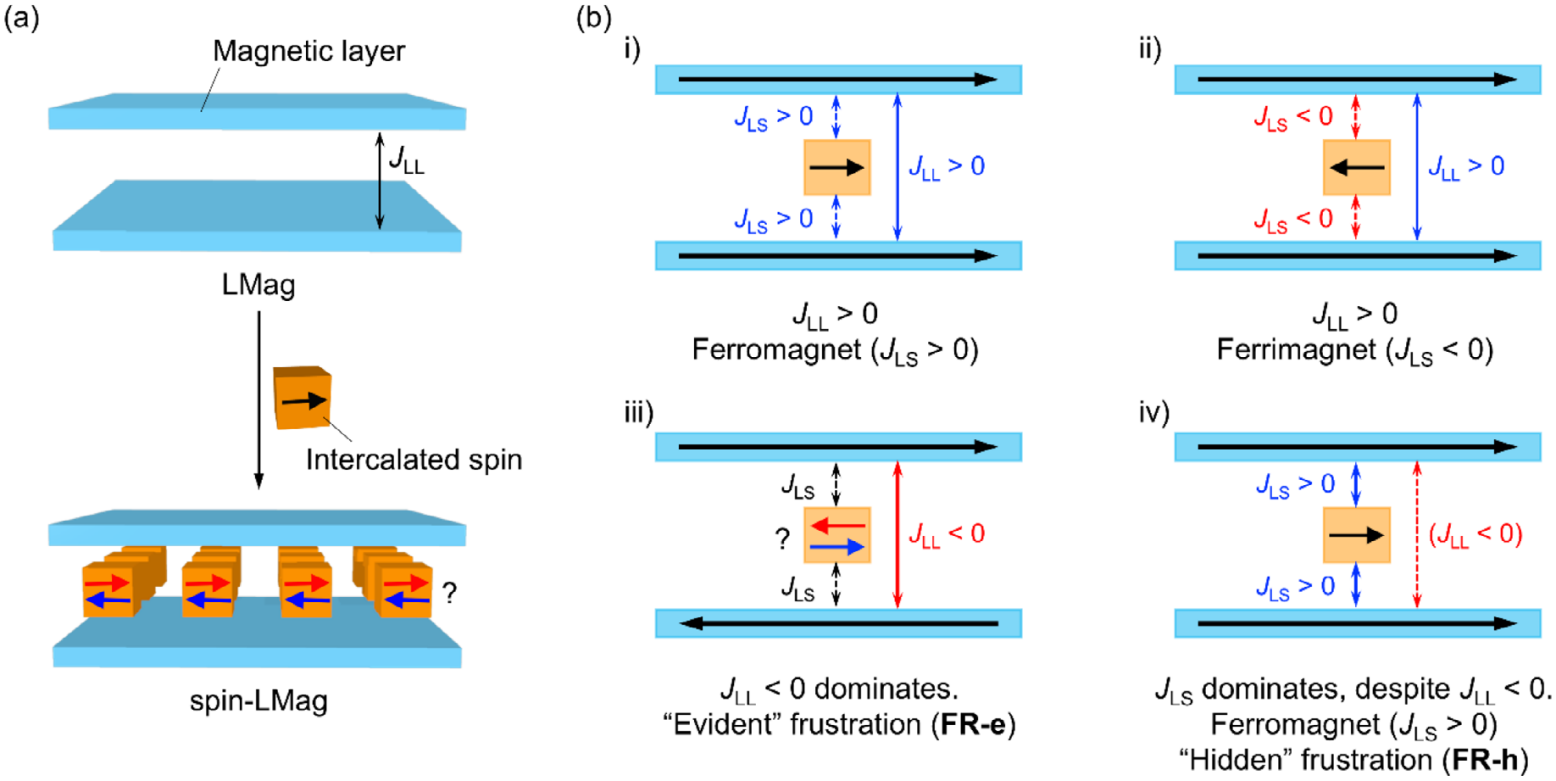
Proposed spin arrangement mechanisms in the present spin‐intercalated layered magnets. The blue‐ and orange‐colored blocks represent the magnetic layers and intercalated spin, respectively. *J*_LL_ and *J*_LS_ are interlayer dipole‐dipole and inserted spin‐layer coupling interactions, respectively.

The magnetic phase transition behavior of layered magnets (LMags) with strong intralayer magnetic correlation is sensitive to their interlayer environment and interlayer intervening paramagnetic species. This makes them an effective platform for constructing magnetic materials that respond to various external stimuli, including chemical stimuli such as mass transport^[^ ^22^, ^23^, ^24^, ^25^, ^26^, ^27^, ^28^, ^29^, ^31^ ^]^ and ion transport.^[^ ^32^, ^33^, ^34^, ^35^ ^]^ Since strong magnetic correlations within a layer produce strong dipole moments, i.e., internal magnetic fields,^[^ ^36^ ^]^ the insertion of paramagnetic spins into the layer to provide spin‐intercalated LMags (spin‐LMags) is affected by both, the interaction between the interlayer dipole moments, i.e., interlayer interaction, *J*_LL_, and the interaction between the dipole moment of each layer and the inserted paramagnetic spins, i.e., *J*_LS_ (**Scheme** **1**a).^[^ ^37^ ^]^ If *J*_LL_ is ferromagnetic (*J*_LL_ > 0), then the system has a magnetic body exhibiting spontaneous magnetization regardless of whether *J*_LS_ is ferromagnetic (F) or antiferromagnetic (AF), producing a ferromagnet with *J*_LS_ > 0 or a ferrimagnet with *J*_LS_ < 0 (mechanisms i) and ii) in Scheme 1b). On the other hand, if *J*_LL_ is AF (*J*_LL_ < 0) and dominant in the system, the system has a spin frustrating (FR) state with competing *J*_LS_, regardless of its sign (mechanisms iii) and iv) in Scheme 1b). The frustration type is further classified into two subtypes depending on the relative magnitude of *J*_LS_ versus *J*_LL_: 1) when |*J*_LS_| ≤ |*J*_LL_|, the effect of spin frustration on the magnetic behavior is “evident,” and the magnetically ordered phase undergoes spin reorientation (mechanism iii) in Scheme 1b, denoted as **FR‐e**), and 2) when |*J*_LS_| >> |*J*_LL_|, the effect of spin frustration on the magnetic behavior is “hidden,” resulting in a behavior akin to only a ferromagnet or ferrimagnet (mechanism iv) in Scheme 1b, denoted as **FR‐h**). Since the *J*_LL_ and *J*_LS_ associated with the spin frustration mechanism are extremely sensitive to structural changes, the fact that they can experience frustration relief when |*J*_LS_| > |*J*_LL_| despite *J*_LL_ < 0 makes spin‐LMags excellent targets for controlling the magnetic phase through the insertion and ejection of guest molecules.

**Scheme 2:**
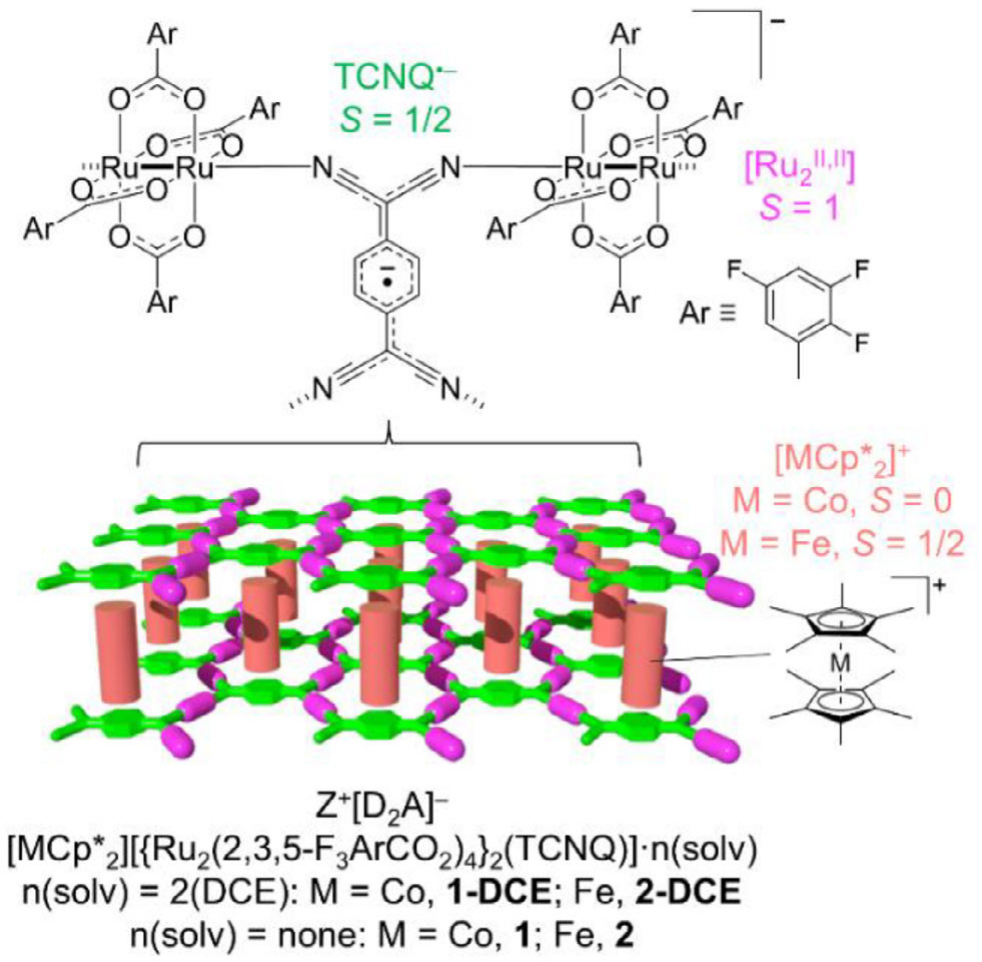
Schematic representations of Z^+^[D_2_A]^–^ type spin‐intercalated layered magnets and component building units.

Here, we demonstrate magnetic phase switching between **FR‐e** and **FR‐h**, associated with the spin frustration mechanism in spin‐LMags, by controlling the guest sorption. The initial frameworks with 1,2‐dichloroethane (DCE) guests are [MCp*_2_][{Ru_2_(2,3,5‐F_3_ArCO_2_)_4_}_2_(TCNQ)]·2DCE (M = Co, **1‐DCE**; Fe, **2‐DCE**), where [MCp*_2_]^+^ = decamethylmetallocenium; 2,3,5‐F_3_ArCO_2_^−^ = 2,3,5‐trifluorobenzoate, which maintain their isostructure when intercalated metal ions (M) are converted (**Scheme** **2**). The [{Ru_2_(2,3,5‐F_3_ArCO_2_)_4_}_2_(TCNQ)]^–^ part forms a strongly correlated ferrimagnetic layer and the [MCp*_2_]^+^ part forms the interlayer‐intercalated molecule that supplies different spins of *S* = 0 and 1/2 for M = Co and Fe, respectively. Compound **1‐DCE**, which contains diamagnetic [CoCp*_2_]^+^, exhibits interlayer AF interactions (*J*_LL_ < 0), forming an antiferromagnet at the Néel temperature (*T*_N_ = 86 K). Compound **2‐DCE**, which features [FeCp*_2_]^+^ with *S* = 1/2, displays a spin frustration phase in a confined area at low applied fields in the temperature range from 94 K (= *T*_N_) to 70 K (= *T*_R_: phase transition temperature between the spin frustration phase and ferromagnetic (F) phase) between paramagnetic (P) and F phases. This occurs due to competing magnetic interactions between *J*_LL_ < 0 and *J*_LS_ > 0, established by the interactions between each layer and the intercalated [FeCp*_2_]^+^ spin. Subsequently, guest‐free compounds **1** and **2** are synthesized by eliminating DCE crystallization solvents while maintaining an isostructural form between them. Compound **1** behaves as a simple antiferromagnet at *T*_N_ = 94 K, whereas **2** transforms into a ferromagnet by two‐step spin long‐range ordering at critical temperatures of *T*_C1_ = 97 K and *T*_C2_ = 92 K owing to exchange frustration relief, attributed to *J*_LS_ surpassing the AF *J*_LL_. These results imply that the spin‐frustrated phase is “hidden” in **2**. Notably, the change in **2‐DCE** and **2**, achieved by tuning the spin frustration phase, is completely reversible upon desolvation/solvation. This is the first time that the nature of spin frustration triggers the reversible switching of the magnetic phase induced by guest accommodation in a spin‐LMag.

## 2 Results and Discussion

### 2.1 Materials

Organic radical molecules can serve as magnetic components in the potential creation of magnetic metal‐organic frameworks (MOFs).^[^ ^38^ ^]^ In this regard, 7,7,8,8‐tetracyano‐*p*‐quinodimethane (TCNQ) and its derivatives (TCNQR) stand out owing to their appropriate size and planarity, promising application as ligands,^[^ ^39^ ^]^ as well as tunable electron‐accepting ability, which allows them to act as electron acceptors (A).^[^ ^40^ ^]^ Assembly reactions with carboxylate‐bridged paddlewheel‐type diruthenium(II, II) components ([Ru_2_^II,II^(RCO_2_)_4_], [Ru_2_^II,II^]) as electron donors (D) in a 2:1 D/A stoichiometry have produced a series of 2D D_2_A‐type layered magnetic MOFs, the electronic and magnetic properties of which strongly depend on charge transfer within the D_2_A layer, as well as interlayer magnetic interactions (*J*_LL_).^[^ ^41^, ^42^ ^]^ The insertion of the cation Z^+^ between D_2_A layers forms Z^+^[D_2_A]^–^ type hybrid layer compounds, denoted as Z^+^–intercalated LMags (Scheme 2).^[^ ^36^, ^43^, ^44^ ^]^ As Z^+^ = [MCp*_2_]^+^, [MCp*_2_][{Ru_2_(2,3,5‐F_3_ArCO_2_)_4_}_2_(TCNQ)]·2DCE (M = Co, **1‐DCE**; Fe, **2‐DCE**) were synthesized from the reaction of [Ru_2_^II,II^(2,3,5‐F_3_ArCO_2_)_4_] with [MCp*_2_] and TCNQ in a layered solution of dichloromethane (DCM) and DCE (see Experimental Section). Solvent‐free compounds **1** and **2** were prepared by evacuating **1‐DCE** and **2‐DCE** crystals at 353 K for 12 h, respectively. The crystallinity of the compounds was well‐maintained after evacuation.

Infrared (IR) and Raman spectra provide crucial indicators of compound formation and the oxidation state of the constituent units. In Figure S1a (Supporting Information), the IR spectra measured at room temperature show *ν*_C≡N_ bands at 2197 and 2156 cm^−1^ for **1‐DCE**, 2194 and 2154 cm^−1^ for **2‐DCE**, 2193 and 2155 cm^−1^ for **1**, and 2196 and 2155 cm^−1^ for **2**. All these bands can be assigned to the formation of the monoanion radical of TCNQ, i.e., TCNQ^•–^, as these bands are observed at 2222 cm^−1^ and at 2209 and 2179 cm^−1^ for TCNQ (original neutral) and Li^+^TCNQ^•−^, respectively. Similarly, the *ν*_C≡N_ bands in the Raman spectra also agree with the TCNQ^•–^ form, displaying small shifts to lower wavenumbers from those of the original neutral TCNQ, albeit of low intensity. These bands are observed at 2219 and 2165 cm^−1^ for **1‐DCE**, 2219, 2203, and 2166 cm^−1^ for **2‐DCE**, 2221 and 2151 cm^−1^ for **1**, and 2221 and 2163 cm^−1^ for **2**, versus their positions at 2223 and 2219 cm^−1^ for TCNQ and Li^+^TCNQ^•–^, respectively (Figure S1b, Supporting Information). In addition, the ν_C = C_ bands in the Raman spectra provide clear evidence that the bands of the four compounds shift to lower wavenumbers compared to that of the pristine compound at 1454 cm^−1^ (TCNQ), to 1370, 1370, 1335, and 1368 cm^−1^ for **1‐DCE**, **2‐DCE**, **1**, and **2**, respectively (Figure S1b, Supporting Information). The aforementioned results imply the presence of TCNQ^•−^ in the four compounds, regardless of their solvation.

### 2.2 Structures and Electronic States

**Figure 1:**
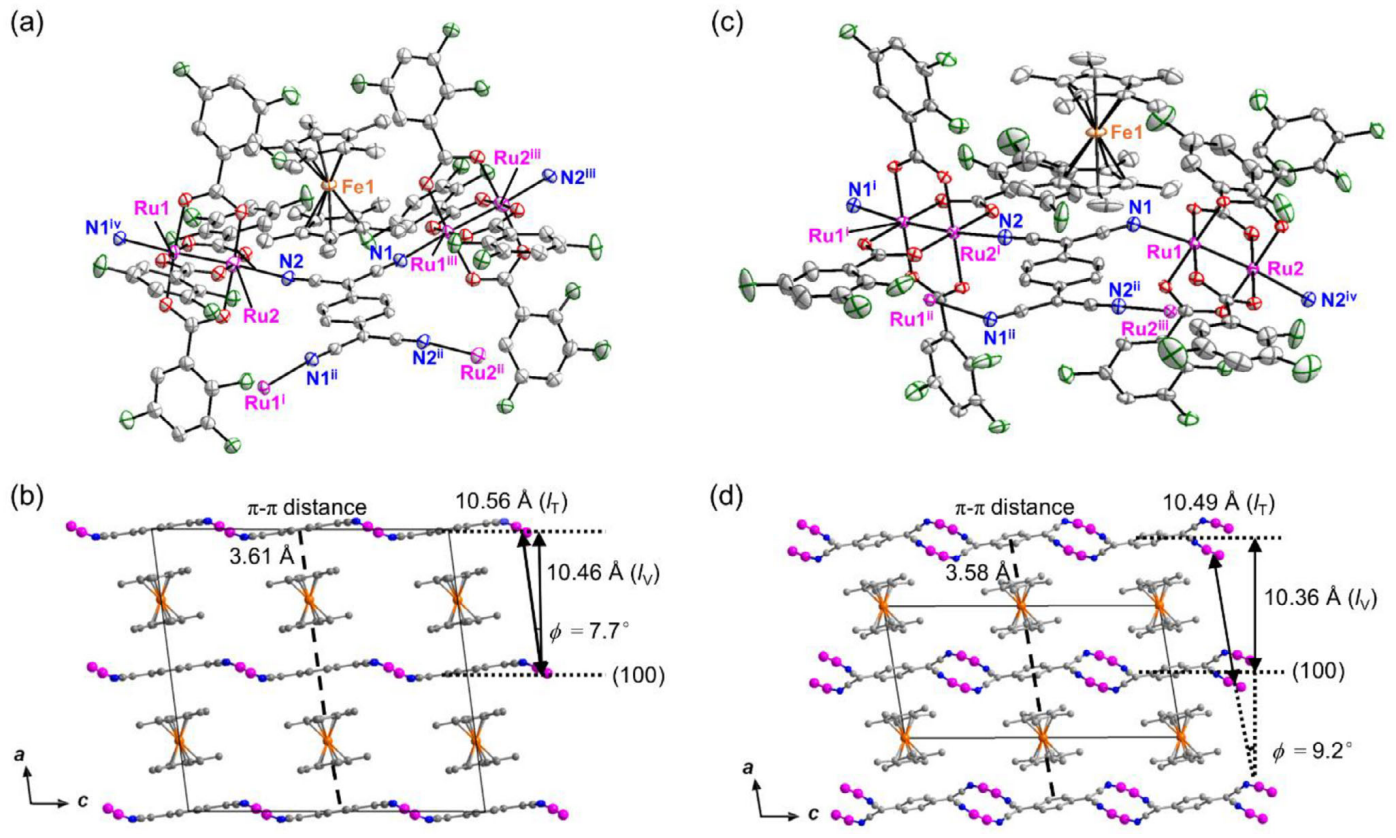
Structures of solvated **2‐DCE** and solvent‐free **2**. a) ORTEP representation of the formula unit and b) packing view along the *b*‐axis for **2‐DCE**, where the disordered Part‐B atoms, the crystallization of DCE, hydrogen atoms, and 2,3,5‐F_3_ArCO_2_^−^ ligands (only in b) around the Ru centers are omitted for clarity. The symmetry operations are (i) *x*, −*y* + 3/2, *z* + 1/2, (ii) −*x* + 1, −*y* + 1, −*z* + 2, (iii) −*x* + 1, *y* − 1/2, −*z* + 3/2, and (iv) −*x* + 1, *y* + 1/2, −*z* + 3/2. c) ORTEP representation of the formula unit and (d) packing view along the *b*‐axis for **2**, where the crystallization of DCE, hydrogen atoms, and 2,3,5‐F_3_ArCO_2_^−^ ligands (only in d) around the Ru centers are omitted for clarity. The symmetry operations are (i) −*x* + 1, *y* − 1/2, −*z* + 1/2, (ii) −*x* + 1, −*y* + 1, −*z* + 1, (iii) *x*, −*y* + 3/2, *z* + 1/2, and (iv) −*x* + 1, *y* + 1/2, −*z* + 1/2. The Ru, Fe, C, O, N, and F atoms are color‐coded as purple, orange, gray, red, blue, and green, respectively.

Structural information obtained using Single‐crystal X‐ray diffraction characterization at 103 K confirmed that **1‐DCE** and **2‐DCE** are isostructural (Table S1, Supporting Information). Powder X‐ray diffraction (PXRD) patterns indicate the purity of the bulk samples (Figure S2a, Supporting Information). The initial compounds were crystallized in the space group *I*2/*a* (monoclinic, No. 15) with an asymmetric unit comprising one whole [Ru_2_] and half of TCNQ and [MCp*_2_], that is, half of the formula unit (**Figure** **1**a, Figures S3a and S4a, Supporting Information). The TCNQ moiety had an inversion center. One two‐fold axis passed through the metal centers of [MCp*_2_], and was related to the two Cp* rings. All the [Ru_2_] atoms were crystallographically unique. A hexagonal fishnet‐like layer was constructed through *µ*_4_‐bridging coordination mode, in which each of the cyano groups from TCNQ bonded in an *η*¹ fashion to the axial sites of [Ru₂]. This fishnet layer lies on the (100) plane with an interlayer vertical (*l*_V_) distance of 10.46 Å for **1‐DCE** and **2‐DCE** (Figure 1b; Figures S3b,S4b,S5, and S6, Supporting Information). The Ru atoms and [MCp*_2_] showed severe intricate positional disorders, which distinguished two types of moieties, Part‐A and ‐B, with occupancies of ≈80% and 20%, respectively (Figures S3 and S4a,b, Supporting Information).^[^ ^45^ ^]^ Considering these position occupancies, the minor position of Part‐B could be present randomly among Part‐A. Accordingly, two interlayer translational distances (*l*_T_) could be defined for Part‐A/‐B (Figures S3b and S4b, Supporting Information), with values of 10.55/10.54 Å for **1‐DCE** and 10.56/10.55 Å for **2‐DCE**. Consequently, the fresh compounds would have two slant angles *ϕ* (= arccos(*l*_V_/*l*_T_)) between the D_2_A layers based on the disordered parts (Figures S3b and S4b, Supporting Information). The *ϕ* values for Part‐A/‐B are 7.4/6.8° for **1‐DCE** and 7.7/7.2° for **2‐DCE**. The presence of slanted layers with a random appearance is the most likely origin for the spin‐canting arrangement in the spin ordering between layers, as shown in the magnetism section for the compounds (vide infra). [MCp*_2_]^+^ are sandwiched between two TCNQ moieties via π‐stacking interactions in the form of {∙∙∙[MCp*_2_]∙∙∙TCNQ∙∙∙} (Figure 1b; Figures S3b and S4b, Supporting Information), as found in reported pillared layer frameworks.^[^ ^44^, ^46^ ^]^ Similarly, two π‐stacking distances between the centers of the Cp* ring and TCNQ C_6_ rings are observed, resulting from the disordered Part‐A/‐B, which are 3.65/3.70 Å for **1‐DCE** and 3.57/3.61 Å for **2‐DCE**. Compounds **1‐DCE** and **2‐DCE** contain two molecules of DCE per formula unit as crystallization solvents on both sides of TCNQ (Figures S5 and S6, Supporting Information). Thermal gravimetric analysis (TGA) indicates that DCE is gradually lost, even when the samples are maintained at room temperature (Figure S7, Supporting Information).

The structures of the solvent‐free compounds **1** and **2** were also characterized by SCXRD and PXRD, which revealed that isostructurality was maintained between **1** and **2** with the π‐stacked pillared layer feature (Figure 1d; Figures S2b and S4d and Table S2, Supporting Information). Both compounds adopt the monoclinic crystal system and crystallize in the space group *P*2_1_/*c*. The asymmetric unit consisted of one whole [Ru_2_] and half of TCNQ and [MCp*_2_], that is, half of the formula unit, similar to the solvated forms; nevertheless, no disordering was observed (Figure 1c; Figure S4c, Supporting Information). An inversion center was located at the center of TCNQ and [MCp*_2_], while all the [Ru_2_] atoms were crystallographically unique. The values of *l*_V_/*l*_T_ were 10.33/10.44 Å for **1** and 10.36/10.49 Å for **2**, shorter than those in the solvated compounds (Figure 1d; Figures S3b and S4b,d, Supporting Information). The slant angles were 8.2° and 9.2° for **1** and **2**, respectively, slightly larger than those of the solvated compounds (Figure 1d; Figure S4d, Supporting Information). The *π*–*π* stacking distances between the centers of TCNQ and [MCp*_2_] were 3.62 and 3.58 Å for **1** and **2**, respectively, which were slightly decreased by desolvation. Meanwhile, the tilt angles (*δ*) of [MCp*_2_] against the D_2_A layer, 75.4° and 77.3° for **1** and **2**, respectively, were slightly decreased compared to those in the solvated compounds (Figure S8, Supporting Information). Thus, the transition from the solvated to solvent‐free form involves a reduction in the interlayer spacing, which induces a notable effect on the magnetic characteristics (vide infra).

The spin arrangement in the framework is directly associated with the oxidation states of the [Ru_2_], TCNQ, and [MCp*_2_] components. The oxidation state of each component can be evaluated based on its local dimensions and geometry. For the [Ru_2_] moiety, the Ru−O_eq_ bond lengths (O_eq_ refers to the carboxylate oxygen atom of [Ru_2_]) are characteristic to the oxidation state, as found in the ranges of 2.06–2.07 Å and 2.02–2.03 Å for the [Ru_2_^II,II^] and [Ru_2_^II,III^]^+^ states, respectively.^[^ ^47^, ^48^ ^]^ For **1‐DCE**, which has one unique Ru_2_ dimer unit composed of Ru(1) and Ru(2) sites, the mean Ru−O_eq_ bond lengths around the Ru(1) and Ru(2) sites are Ru(1)/Ru(2) = 2.069(2)/2.071(2) Å in Part‐A and 2.085(4)/2.082(4) Å in Part‐B; for **2‐DCE**, the mean Ru−O_eq_ bond lengths are Ru(1)/Ru(2) = 2.066(2)/2.066(2) Å in Part‐A and 2.080(5)/2.066(3) Å in Part‐B, revealing that all [Ru_2_] dimers correspond to the [Ru_2_^II,II^] state regardless of their disordered parts (Table S3, Supporting Information). The charge transfer states (*ρ*) of the TCNQ moiety, which can be estimated using the Kistenmacher relationship,^[^ ^49^, ^50^, ^51^ ^]^ are –1.19(10) in **1‐DCE** and –1.47(13) in **2‐DCE** (Table S4, Supporting Information), implying the existence of the monoanion state, TCNQ^•−^. The oxidation state of [MCp*_2_]*^n^* ^+^ (*n* = 0, 1) can be approximated from the M−Cp*_cent_ distance (Cp*_cent_ = center of the Cp* ring), for which the referred M−Cp*_center_ distances for M = Co^II^, Co^III^, Fe^II^, and Fe^III^ are reportedly ≈ 1.71, 1.65–1.66, 1.66, and 1.69–1.71 Å, respectively.^[^ ^52^, ^53^, ^54^, ^55^, ^56^ ^]^ The observed M−Cp*_cent_ distances for Part‐A/‐B are 1.65/1.59 Å and 1.70/1.72 Å in **1‐DCE** and **2‐DCE**, respectively (Table S5, Supporting Information). These results suggest that the [MCp*_2_]^n+^ moieties in both compounds have a mono‐cationic state [M^III^Cp*_2_]^+^. Consequently, one‐electron transfer occurs from neutral [M^II^Cp*_2_] to TCNQ in the assembly process, leading to the charge distribution of [M^III^Cp*_2_]^+^[{Ru_2_^II,II^}_2_(TCNQ^•−^)].

The electronic states of the solvent‐free compounds **1** and **2** were deduced using the same method (Tables S3–S5, Supporting Information). The overall electronic structures of **1‐DCE** and **2‐DCE** remained unchanged in **1** and **2**, respectively, which can be described as [M^III^Cp*_2_]^+^[{Ru_2_^II,II^}_2_(TCNQ^•−^)]. These structural observations, along with the presence of TCNQ^•–^, are in full agreement with the spectral data (Figure S1, Supporting Information).

### 2.3 Magnetic Properties of 1‐DCE

**Figure 2:**
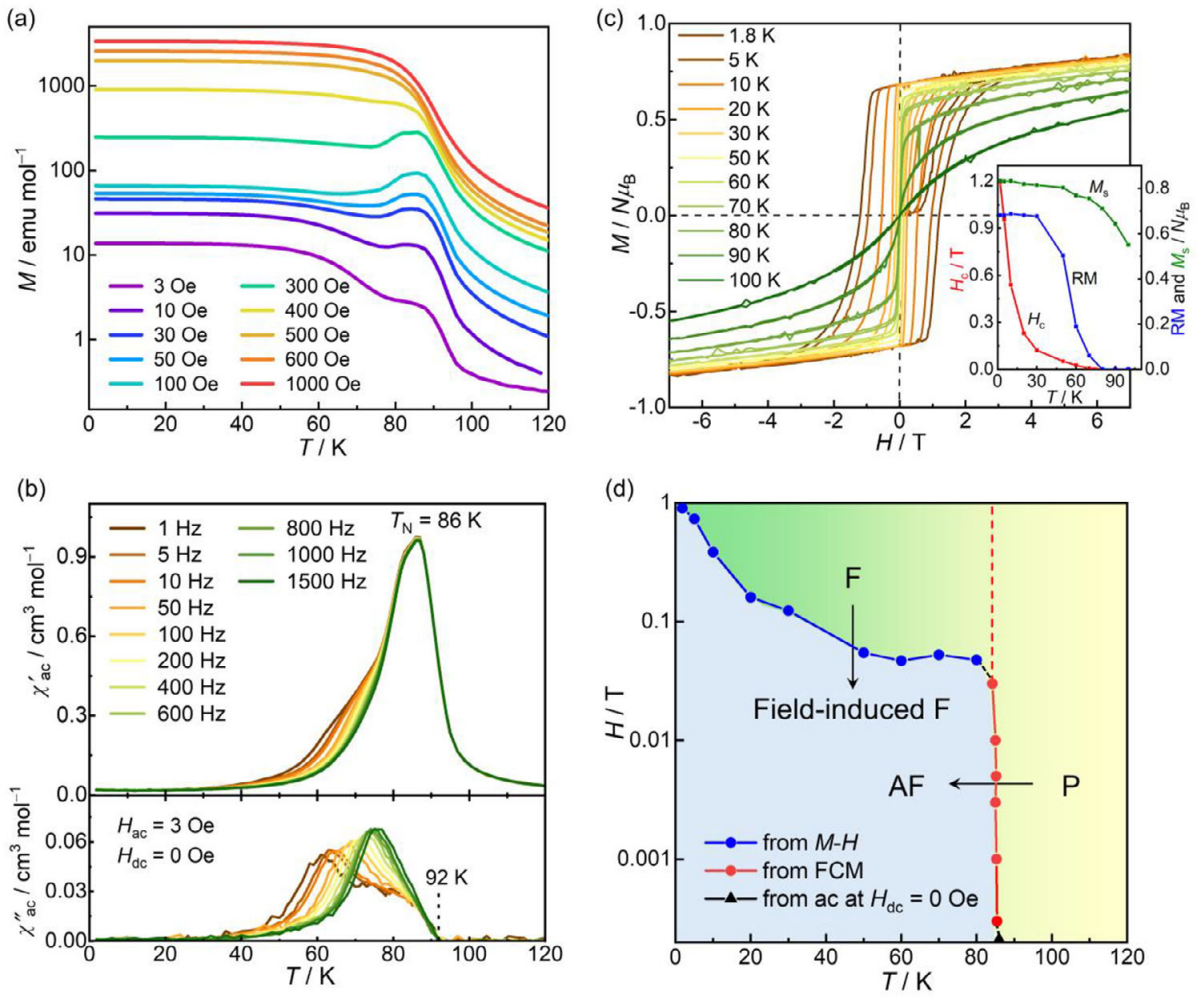
Magnetic properties of **1‐DCE**. a) Field‐cooled magnetization curves collected under different dc external fields in the 3 Oe – 1 kOe range. b) Temperature dependence of the ac magnetic susceptibilities (*χ*', in‐phase; *χ*", out‐of‐phase) at a zero dc field and 3 Oe oscillating field. c) Field dependence of the magnetization measured at various temperatures between 1.8 and 100 K. The inset shows the temperature dependence of the coercive field (*H*_c_), remnant magnetization (RM), and saturated magnetization (*M*_s_) at 7 T. d) *H*–*T* phase diagram, in which AF, F, and P denote antiferromagnetic, ferromagnetic, and paramagnetic phases, respectively.

**1‐DCE** can be regarded as a normal layered magnetic system because it has diamagnetic [CoCp*_2_]^+^ between the layers. Therefore, clarifying the magnetic behavior of this compound will provide knowledge about the magnetic interaction *J*_LL_ between the layers, which can serve as a prototype for understanding the effect of spin insertion in **2‐DCE**. The field‐cooled magnetizations (FCMs; *M*) of **1‐DCE** were collected at several dc fields (*H*_dc_) (**Figure** **2**a); profiles of the magnetic susceptibility (*χ* = *M*/*H*_dc_) and *χT* product acquired at *H*_dc_ = 1 kOe are provided in Figure S9a (Supporting Information) and will be discussed subsequently. The value of *χT* at 300 K is 2.08 cm^3^ K mol^−1^. This is smaller than the calculated value of 2.38 cm^3^ K mol^−1^ expected from the sum of individual paramagnetic units: 2 × [Ru_2_^II,II^] with *S* = 1 and 1 × TCNQ^•−^ with *S* = 1/2 as *g*_Ru_ = *g*_Rad_ = 2.00. This discrepancy is attributed to the strong AF coupling (*J*_intra_ < 0) between [Ru_2_^II,II^] and TCNQ^•–^ in the [{Ru_2_^II,II^}_2_TCNQ]^−^ layer. As the temperature decreases, the *χT* value gradually increases, then suddenly increases at ≈ 100 K to reach a maximum of 197 cm^3^ K mol^−1^ at 69 K, followed by a rapid decrease to 6.27 cm^3^ K mol^−^^1^ at 1.8 K (Figure S9a, Supporting Information). The *χ*–*T* curve also shows a rapid increase at ≈ 100 K, suggesting the onset of long‐range magnetic ordering; thereafter, there is a continuous increase without a decrease until the temperature reaches 1.8 K, resembling the behavior of a normal layered [Ru_2_]_2_TCNQ ferromagnet.^[^ ^57^, ^58^ ^]^ However, the FCM data measured at *H*_dc_ ≤ 300 Oe show a cusp at ≈ 85 K, indicating convergence to the AF ground state (Figure 2a; Figure S10, Supporting Information show the remnant magnetization (RM) and zero‐field‐cooled magnetization (ZFCM), in addition to the FCM).^[^ ^57^, ^59^, ^60^ ^]^ To obtain detailed information about the spin ordering, the ac susceptibilities (*χ′*: in‐phase, *χ′′*: out‐of‐phase) were measured under a zero dc field and an oscillating field of 3 Oe in the frequency range of 1 Hz to 1.5 kHz (Figure 2b). *χ′* shows a frequency‐independent sharp peak at 86 K, which corresponds to the Néel temperature (*T*_N_) for an antiferromagnet, as realized from the cusp in the FCM data at low dc fields. Meanwhile, a significant signal of *χ′′* was observed near *T*_N_, indicating the occurrence of spontaneous magnetization. The appearance of the *χ′′* signal in the AF system indicates that the spin‐canting mode has occurred. In the structural section, **1‐DCE** exhibits two types of disordered positions, Part‐A and ‐B occupying ca. 80:20%, providing different slant angles. The structural disorder leads to a mismatch in the antiferromagnetically coupled spins between the layers, producing a spin‐canting domain response to ac frequencies. This disorder also induces significant domain wall motion, leading to the frequency‐dependent *χ′′*. This frequency dependence was evaluated using the critical scaling approach model employed for common spin glasses;^[^ ^61^, ^62^ ^]^ however, it did not fall within the parameter range of what would be described as a common spin glass (Figure S11, Supporting Information). The relaxation time of the present system was found to be much slower, ≈ 10^−6^ s, than the relatively fast (*τ*_0_ ∼ 10^−12^ – 10^−13^ s) relaxation times of typical spin glasses.^[^ ^63^ ^]^ This is possibly due to the large magnetic domains formed and the large magnetic anisotropy that leads to a high viscosity of the magnetic domains in this layer system.

Metamagnetic behavior was also observed in the field dependence of the magnetization (*M*−*H*) collected in the field range of −7 to 7 T at several temperatures (Figure 2c). Further, the magnetization variation in the initial field sweep from 0 to 7 T underwent a spin flip at a flipping field *H*_ex_, which was determined by the d*M*/d*H*_dc_ plot (Figure S12, Supporting Information). Finally, an *H*−*T* phase diagram for **1‐DCE** was constructed (Figure 2d), which is a typical diagram for metamagnetism. Notably, after achieving the P phase upon applying a high field, **1‐DCE** maintained the spin‐parallel arrangement even at low temperatures below *T*_N_, exhibiting field‐induced ferromagnetism (See Supporting Information) with an RM of 0.68 *Nμ*_B_ and a large coercivity (*H*_c_) of 1.2 T at 1.8 K (inset of Figure 2c); these results are consistent with previous results for [Ru_2_]_2_TCNQ layered antiferromagnets.^[^ ^57^, ^59^, ^60^ ^]^ It is important to note that we uniformly refer to the state in which layer dipoles are aligned in parallel as ferromagnet (F) in this paper, although the hetero‐spin magnetic layer is ordered in a ferrimagnetic spin arrangement as mentioned above (some of previously reported papers have referred it as Ferrimagnet (Fi).^[^ ^22^, ^23^, ^24^, ^25^, ^26^, ^27^, ^31^, ^32^, ^34^, ^36^, ^41^, ^42^, ^58^, ^60^ ^]^ The *H*_c_ value quasi‐exponentially decreased with increasing temperature and finally disappeared at ≈ 80 K, corresponding to *T*_N_ (inset of Figure 2c). These results for **1‐DCE** align well with the electronic states identified through structural and spectroscopic studies.

### 2.4 Magnetic Properties of 2‐DCE

**2‐DCE** is isostructural to **1‐DCE**, but has an intercalated anisotropic spin *S* = 1/2 on [FeCp*_2_]^+^ between the ferrimagnetic layers. The *χT* value at 300 K (*H*_dc_ = 1 kOe) was 2.66 cm^3^ K mol^−1^ (Figure S9b, Supporting Information), which was smaller than the theoretical paramagnetic value of 3.10 cm^3^ K mol^−1^: 2 × [Ru_2_^II,II^] with *S* = 1, 1 × TCNQ^•−^ with *S* = 1/2, and 1 × [FeCp*_2_]^+^ with *S* = 1/2 as *g*_Ru_ = *g*_Rad_ = 2.00 and *g*_Fe_ = 2.80,^[^ ^52^, ^64^ ^]^ because of strong intralayer AF coupling. Upon decreasing the temperature, *χT* gradually increased and then suddenly increased at ≈ 100 K to reach a maximum of 304 cm^3^ K mol^−1^ at 82 K, before rapidly dropping to 3.86 cm^3^ K mol^−1^ at 1.8 K (Figure S9b, Supporting Information). The *χ*−*T* curve of **2‐DCE** shows a similar trend to that of **1‐DCE**, with a rapid increase at ≈ 100 K down to 50 K; however, below 20 K, *χ* decreases due to the contribution of the [FeCp*_2_]^+^ spin.^[^ ^65^, ^66^ ^]^ Since the spins of TCNQ^•−^ and [Ru_2_^II,II^] are antiferromagnetically coupled in the layer, the contribution of the spin‐orbit coupling of [FeCp*_2_]^+^, which is spin‐ferromagnetically coupled with TCNQ^•−^, decreases the net magnetization.^[^ ^44^ ^]^

**Figure 3:**
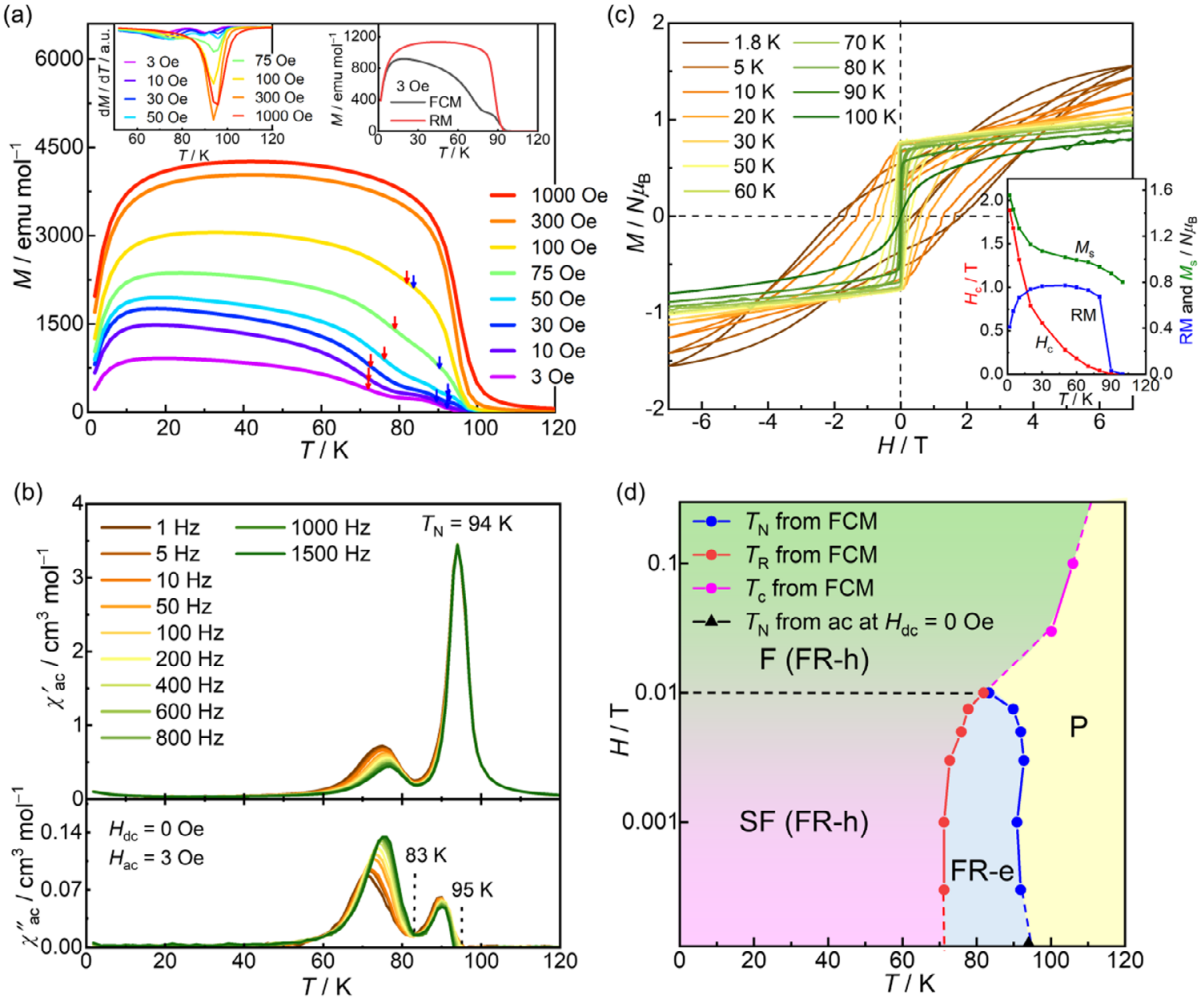
Magnetic properties of **2‐DCE**. a) Field‐cooled magnetization curves recorded under different dc external fields in the 3 Oe – 1 kOe range, where the red and blue arrows represent the spin reorientation (*T*_R_) and AF transition (*T*_N_) temperatures, respectively. The left inset shows the d*M*/d*T* plots based on the FCM curves. The right inset shows the FCM‐RM curves from 120 to 1.8 K under an *H*_dc_ of 3 Oe. b) Temperature dependence of the ac magnetic susceptibilities (*χ*', in‐phase; *χ*", out‐of‐phase) at a zero dc field and 3 Oe oscillating field. c) Field dependence of the magnetization measured at various temperatures between 1.8 and 100 K. The inset shows the temperature dependence of *H*_c_, RM, and *M*_s_ at 7 T. d) *H*–*T* phase diagram, where P, FR‐e, FR‐h, SF, and F denote paramagnetic, “evident” frustrated, “hidden” frustrated, spin‐flopping, and ferromagnetic phases, respectively.

The interlayer interaction *J*_LL_ for **2‐DCE** is expected to be AF, as is the case for **1‐DCE**, which has an isostructural feature. Meanwhile, the [FeCp*_2_]^+^ spin with *S* = 1/2 interferes with the magnetic coupling between the [{Ru_2_}_2_TCNQ]^−^ layers, inducing spin frustration (see mechanism iii) in Scheme 1b). The FCM curves of **2‐DCE** measured at several dc fields (**Figure** **3**a; Figure S13, Supporting Information show the RM, ZFCM, and FCM) are different from those of **1‐DCE**. This difference is particularly pronounced at weak dc fields of less than 75 Oe, at which a two‐step increase in the magnetization is observed. The inflection points of these steps shift depending on the applied fields (Figure 3a), as defined by the d*M*/d*T* plots of the FCM curves (left inset in Figure 3a; Figure S14, Supporting Information), which provide an *H*−*T* phase diagram (Figure 3d). For example, at *H*_dc_ = 3 Oe, the magnetization increases at ≈ 95 K, corresponding to *T*_N_, forming a hilltop, and then increases again at ≈ 75 K (*T*_R_). The subsequent growth of magnetization implies the occurrence of spin reorientation to a new magnetic ordered phase associated with significant coupling with the intercalated spin of [FeCp*_2_]^+^. Thus, this two‐step transition is evidence of spin‐exchange frustration occurring as **FR‐e** in mechanism iii) of Scheme 1b, as was similarly observed in [FeCp*_2_][{Ru_2_(2,3,5,6‐F_4_ArCO_2_)_4_}_2_(TCNQ)] under the same conditions as *J*_LL_ < 0.^[^ ^44^ ^]^ The phase appearing in the temperature range from *T*_N_ to *T*_R_ corresponds to a spin frustration (FR) phase, in which the direction of the [FeCp*_2_]^+^ spin is undecidable between the antiferromagnetically coupled magnetic layers with *J*_LL_ < 0; this limitation is overcome at higher applied fields of *H*_dc_ > 100 Oe (Figure 3a). In the phase below *T*_R_, the *J*_LS_ coupling, which is ferromagnetic between [FeCp*_2_]^+^ and TCNQ^•–^,^[^ ^44^ ^]^ dominates the *J*_LL_ interaction, resulting in frustration relief to merge finally to a F long‐range ordering phase (F) (mechanism iv) in Scheme 1b). Meanwhile, the exchange frustration is sustained even for the F phase in the range below *T*_R_, as if it was “hidden” in the balance of |*J*_LS_| > |*J*_LL_| as *J*_LL_ < 0 (Figure 3d). Notably, the RM curve shows a larger magnetization than the FCM curve when applying weak fields of *H*_dc_ ≤ 100 Oe, and the magnetization disappears at approximately *T*_N_ (right inset in Figure 3a; Figure S13a−d, Supporting Information).^[^ ^31^, ^44^ ^]^ This is the typical behavior of a field‐induced ferromagnet;^[^ ^31^, ^44^ ^]^ on the other hand, the reversed dissociation of the FCM and RM indicates that exchange frustration persists even at temperatures below *T*_R_, i.e., a spin‐flopping (SF) phase (pink area in Figure 3d). However, all spin directions, including [FeCp*_2_]^+^, are fully polarized and converge purely into the F phase under a magnetic field of *H*_dc_ > 100 Oe and at a low temperature, such as 1.8 K (green area in Figure 3d).

Figure 3b shows the ac susceptibility measured under a zero dc field and an oscillating field of 3 Oe. *χ′* shows frequency‐independent sharp peaks, indicating that *T*_N_ = 94 K, even though it involves *χ′′*. The appearance of *χ′′* signals at approximately *T*_N_ results from the presence of the FR phase, which is associated with the spins of [FeCp*_2_]^+^, as well as with the mode of spin canting in the same manner as **1‐DCE**. The frequency‐dependent peaks in both *χ′* and *χ′′* at approximately *T*_R_ near 80 K could be attributed to the presence of the SF mode, which converges to the F phase.^[^ ^44^ ^]^ With increasing applied ac frequency, both peaks below 80 K shift to higher temperatures, which is typically akin to spin‐glass or superparamagnetic behavior. The shift parameter *φ* = (Δ*T*_P_/*T*_P_)/Δ(log*f*) (*T*_P_ is the temperature of the *χ′* cusp; *f* is the applied frequency) of the *χ′* peak is 0.0082, which precisely falls into the general range (0.01 − 0.1) of spin‐glass behavior.^[^ ^67^, ^68^ ^]^ To investigate the details of the spin‐glassy relaxation, a dynamic analysis was conducted based on the conventional power‐law divergence of critical slowing down: *τ* = *τ*_0_(*T*_B_/*T*_SG_ − 1)^−^ *^zv^*, where *τ* = 1/(2π*f*) represents the relaxation time at a frequency *f*, *τ*_0_ is the characteristic relaxation time of the system, *T*_SG_ is the spin‐glass transition temperature as *f* is extrapolated to zero, *zv* is the dynamic critical exponent, and *T*_B_ is the blocking temperature, which is defined as the peak temperature in *χ′′*−*T* plots (Figure S15, Supporting Information).^[^ ^61^, ^62^ ^]^ The fitting result yields *τ*_0_ = 1.1 × 10^−12^ s, *zv* = 7.3, and *T*_SG_ = 73.6 K, close to *T*_R_, indicating a spin‐glassy spin ordering (Figure S16, Supporting Information).^[^ ^63^ ^]^ The frequency dependence of **2‐DCE** is very different from that of **1‐DCE**. This observation provides strong evidence that the spin ordering of [FeCp*_2_]^+^ is involved in the magnetization relaxation near *T*_R_ in **2‐DCE**.

The contribution of [FeCp*_2_]^+^ anisotropic spins is also observed in the *M–H* curve, revealing a “gourd‐shaped” magnetic hysteresis phenomenon below 20 K, where the dipole spins in the magnetically ordered layer and [FeCp*_2_]^+^ spins undergo individual reversals for magnetic field application (Figure 3c).^[^ ^44^ ^]^ The RM and *H*_c_ values of **2‐DCE** are 0.41 *N*μ_B_ and 1.88 T at 1.8 K, respectively (inset of Figure 3c). The magnetic measurement results for **2‐DCE** align well with the electronic states identified through structural and spectroscopic studies.

### 2.5 Magnetic Properties of Guest‐Free 1

**Figure 4:**
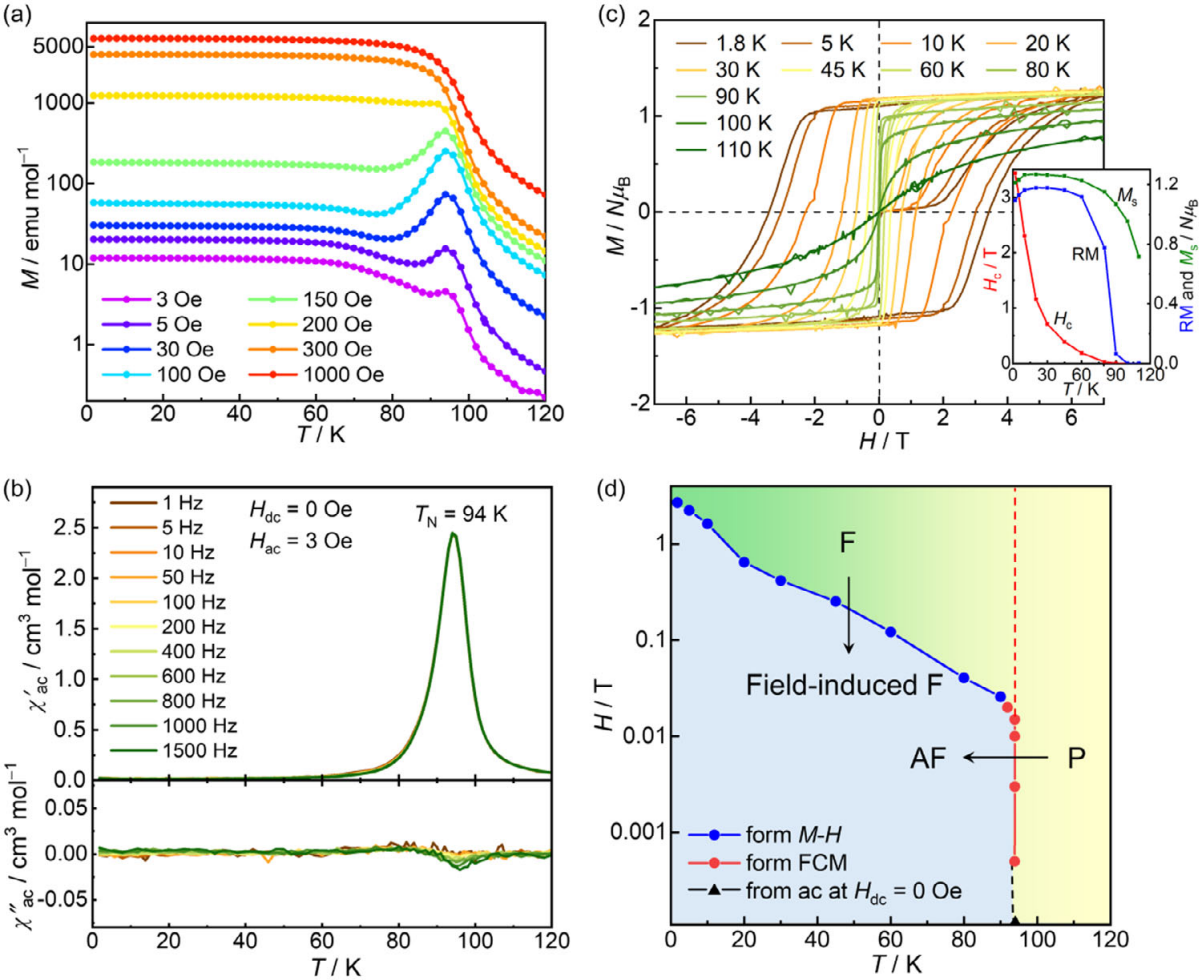
Magnetic properties of guest‐free **1**. a) Field‐cooled magnetization curves collected under different dc external fields in the 3 Oe – 1 kOe range. b) Temperature dependence of the ac magnetic susceptibilities (*χ*', in‐phase; *χ*", out‐of‐phase) at a zero dc field and 3 Oe oscillating field. c) Field dependence of the magnetization measured at various temperatures between 1.8 and 110 K. The inset shows the temperature dependence of *H*_c_, RM, and *M*_s_ at 7 T. d) *H*–*T* phase diagram, in which AF, F, and P denote antiferromagnetic, ferromagnetic, and paramagnetic phases, respectively.

Even after desolvation from **1‐DCE**, **1** remained a typical antiferromagnet, that is, the interlayer dipole interaction *J*_LL_ was AF. The FCM curve of **1** at *H*_dc_ = 1 kOe is shown in Figure S17a (Supporting Information). The *χT* value at 300 K is 2.05 cm^3^ K mol^−1^, which also implies strong AF coupling (*J*_intra_ < 0) between the spins in the [{Ru_2_^II,II^}_2_TCNQ]^−^ layers, as observed in **1‐DCE**. At ≈ 100 K, the *χT* profile exhibits a steep increase, with a maximum value of 420 cm^3^ K mol^−1^ at 80 K, followed by a decrease to 11.5 cm^3^ K mol^−1^ at 1.8 K, indicating the occurrence of long‐range ordering similar to that in **1‐DCE**. **Figure** **4**a shows the FCM curves measured at several dc fields (Figure S18, Supporting Information shows the RM, ZFCM, and FCM). The FCM at *H*_dc_ ≤ 150 Oe exhibits a typical Λ‐shaped feature with a cusp at 94 K, revealing the AF order of [{Ru_2_^II,II^}_2_TCNQ]^−^ layers and the occurrence of spin‐flipping above *H*_dc_ > 200 Oe. The ac susceptibility measurement also confirms the AF ground state; *χ*′ shows a sharp peak at *T*_N_ = 94 K without frequency dependence, while there is no anomaly in *χ*′′ at *T*_N_ (Figure 4b). This difference from the *χ′′* of **1‐DCE** indicates that the structure of **1** is undisturbed by the structural disordering seen in **1‐DCE**. Spin‐flipping is also detected in the *M*−*H* curves measured at several temperatures; *H*_ex_ is determined from the d*M*/d*H* plots of each initial magnetization process (Figure 4c; Figure S19, Supporting Information). The *H*−*T* phase diagram shows a typical case of metamagnetism of **1** (Figure 4d). Notably, the characteristics of a higher *T*_N_ and larger *H*_c_ in **1** than those in **1‐DCE** imply that AF *J*_LL_ in **1** is stronger than that in **1‐DCE**, which is in agreement with their structural characteristics of a shorter *l*_V_ and *l*_T_ in **1**.^[^ ^24^ ^]^

Similar to **1‐DCE**, after achieving a F phase on applying a high field at low temperatures below *T*_N_, **1** maintains a F phase, exhibiting field‐induced ferromagnetism (See Supporting Information) with an RM of 1.1 *Nμ*_B_ and a large *H*_c_ of 3.4 T at 1.8 K (Figure 4c inset).^[^ ^57^, ^59^, ^60^ ^]^ With increasing temperature, the *H*_c_ value decreases in a quasi‐exponential manner, and finally vanishes near 90 K, corresponding to *T*_N_ (inset of Figure 4c). All the above results of magnetic measurements for **1** well agree with the electronic states assigned by the structural and spectroscopic studies.

### 2.6 Magnetic Properties of Guest‐Free 2

Figure S17b (Supporting Information) shows the FCM curve for **2** at *H*_dc_ = 1 kOe. The *χ* and *χT* profiles exhibit similar trends to those in the solvated **2‐DCE**. The *χT* of 2.34 cm^3^ K mol^−1^ at 300 K increases suddenly to the maximum of 411 cm^3^ K mol^−1^ at 80 K, then decreases to 2.8 cm^3^ K mol^−1^ at 1.8 K. *χ* also increases at ≈ 80 K and decreases below 20 K owing to the effect of the spin‐orbit coupling of [FeCp*_2_]^+^, which is ferromagnetically coupled with the TCNQ^•–^ moieties. Because **2** is isostructural with **1**, the *J*_LL_ < 0 property should remain in **2**. Furthermore, as expected from the behavior of **1**, the magnitude of *J*_LL_ in **2** is expected to be higher than that in **2‐DCE**. Therefore, the spin‐exchange frustration mode can be expected in **2**. Nevertheless, the overall behavior of **2** is akin to that of ferromagnets, and *J*_LL_ behaves as though ferromagnetic. This is because the contribution of *J*_LS_ outweighs that of *J*_LL_ in **2**.

**Figure 5:**
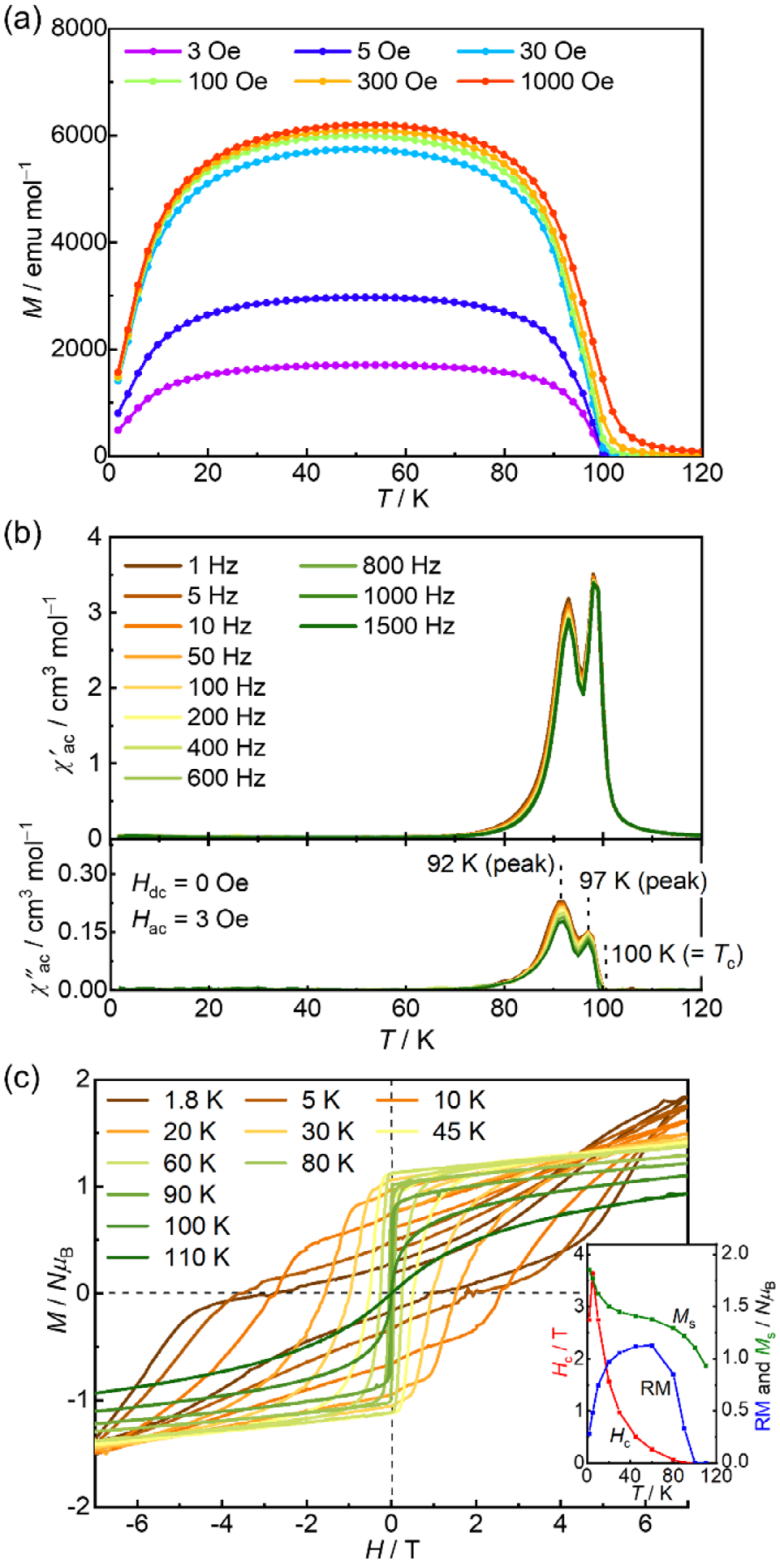
Magnetic properties of guest‐free **2**. a) Field‐cooled magnetization curves collected under different dc external fields in the 3 Oe – 1 kOe range. b) Temperature dependence of the ac magnetic susceptibilities (*χ*', in‐phase; *χ*", out‐of‐phase) at a zero dc field and 3 Oe oscillating field. c) Field dependence of the magnetization measured at various temperatures between 1.8 and 110 K. The inset shows the temperature dependence of *H*_c_, RM, and *M*_s_ at 7 T.

**Figure** **5**a shows the FCM curves measured at several weaker dc fields for **2**. The magnetization exhibits a monotonic increase until 60 K, showing mere F behavior (Figure S20, Supporting Information shows the RM, ZFCM, and FCM). This behavior is similar to that observed in [CrCp*_2_][{Ru_2_(2,3,5,6‐F_4_ArCO_2_)_4_}_2_(TCNQ)], where a larger spin *S* = 3/2 of [CrCp*_2_]^+^ is sandwiched between Fi layers by changing from [FeCp*_2_]^+^ with *S* = 1/2 that provides an exchange frustration system.^[^ ^44^ ^]^ Thus, **2** is an **FR‐h** system, as provided in mechanism iv) in Scheme 1b. Unlike **2‐DCE**, the contribution of *J*_LS_ > 0 exceeds that of *J*_LL_ < 0; thus, the nature of the magnetic ordering is visually determined by the contribution of *J*_LS_, although *J*_LL_ is still AF. Consequently, the FR phase is relieved in **2** as a “hidden” frustration phase, **FR‐h**.

To investigate the long‐range order in detail, the ac susceptibility was measured at *H*_dc_ = 0 Oe and *H*_ac_ = 3 Oe (Figure 5b). Upon cooling from 120 K, a cusp appeared in the *χ′* profile at 98 K without noticeable frequency dependence, accompanied by a raise in *χ*′′ at 97 K. As the temperature decreased, the second peak without frequency dependence in both *χ′* and *χ*′′ appeared at 93 and 92 K, respectively. The occurrence of two peaks in *χ*′′ implied stepwise long‐range magnetic orders with a spontaneous magnetization at transition temperatures of 97 K (*T*_C1_) and 92 K (*T*_C2_). At *T*_C1_, the magnetic order was established by functioning as a quasi‐F interlayer interaction *J*_LL_ with short‐range F interaction *J*_LS_. At *T*_C2_, the anisotropic spins of [FeCp*_2_]^+^ were incorporated into the magnetic long‐range ordering (Figure S21, Supporting Information). The effect of the tight binding of [FeCp*_2_]^+^ between the ferrimagnetic layers in **2** was observed in the magnetic hysteresis loops (Figure 5c). The hysteresis feature at 1.8 K, with RM = 0.28 *N*μ_B_ and *H*_c_ = 2.7 T, was more significantly deformed than that of **2‐DCE** (RM = 0.41 *N*μ_B_ and *H*_c_ = 1.88 T) (inset of Figure 5c). The larger *H*_c_ value in **2** than that in **2‐DCE** indicates that the spin of [FeCp*_2_]^+^ was more strongly fixed anisotropically between the ferrimagnetic layers. The results from the magnetic measurements for **2** are in strong agreement with the electronic states determined by structural and spectroscopic analyses.

### 2.7 Reversible Switching between FR‐e and FR‐h via Solvation Cycles

**Figure 6:**
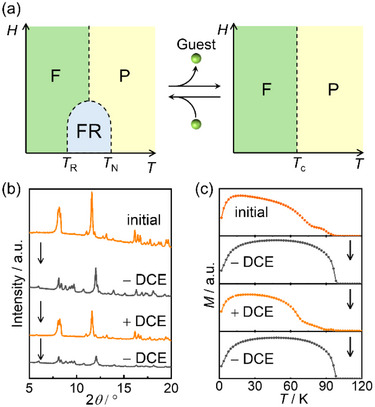
a) Reversible switching between **FR‐e** (left) and **FR‐h** (right) systems. b) PXRD patterns and c) field‐cooled magnetizations under a dc field of 10 Oe for the solvated phase (**2‐DCE**, orange curves) and solvent‐free phase (**2**, gray curves) in the desolvation/solvation cycles.

By changing the inserted spins into the layered antiferromagnet and its structural environment, phase transformation can be achieved using the stress/relief of spin‐exchange frustration. In this study, variations in the structural environment were achieved by removing crystallization solvents (DCE). In other words, the desorption and adsorption of DCE act as triggers for the change between **FR‐e** in **2‐DCE** and **FR‐h** in **2** (**Figure** **6**a). Compound **2‐DCE** loses two molar crystalline DCE molecules by evacuating at 353 K for 12 h, forming guest‐free **2** (it is also possible to achieve the same between **1‐DCE** and **1**; see Figure S22, Supporting Information). To explore the reversibility of this system, crystals of **2** were exposed to DCE vapor under an N_2_ atmosphere at room temperature. The desolvated compound **2** slowly adsorbed DCE molecules and reverted completely to the solvated phase **2‐DCE** after one week, as shown in the PXRD patterns (Figure 6b). Magnetic measurements of **2‐DCE** and **2** were conducted using the sample, for which the aforementioned desolvation/solvation treatments were applied (ex situ sampling); the FCM at *H*_dc_ = 10 Oe was measured at each solvation/desolvation treatment (Figure 6c). The magnetic behaviors of **2‐DCE** and **2** were repeatedly reproduced, indicating the successful switching of two spin‐frustrated systems **FR‐e** and **FR‐h** in **2‐DCE** by DCE guest accommodation, i.e., switching between mechanisms iii) and iv) in Scheme 1b.

After desolvation, an increase in the AF transition temperature (Δ*T*_N_ = 8 K for **1‐DCE** and **1**) was observed, implying an enhanced |*J*_LL_| in guest‐free **1**. Although the *T*_N_ of **2** cannot be extracted due to the “hidden” *J*_LL_, the situation is the same for **2‐DCE** and **2**, that is, **2** has a larger |*J*_LL_|. Meanwhile, the several distances, such as *l*_V_, *l*_T_, and the one between the C_6_ ring of TCNQ and the center of Cp*, all decrease in **2**; thus, **2** is expected to have a larger |*J*_LS_| as well as |*J*_LL_|. To establish a “hidden” FR environment, |*J*_LS_| should be significantly enhanced compared to |*J*_LL_|. This may be ascribed to the smaller tilted angle *δ* between the D_2_A layer and [FeCp*_2_]^+^ (Figure S7, Supporting Information); a smaller *δ* of **2** implies a higher |*J*_LS_| because the directions of the magnetic easy‐axes of [FeCp*_2_]^+^ and magnetic anisotropy of the D_2_A layer are more consistent.^[^ ^36^, ^69^ ^]^ These facts agree with the change from **FR‐e** in **2‐DCE** to **FR‐h** in **2**. Although the structural change accompanied by desolvation is small, it significantly alters the balance of magnetic interactions, triggering a switch in the exchange frustration state.

## 3 Conclusion

Stimuli adjustment of the inherent magnetic interactions within low‐dimensional magnetic systems is a crucial tool for developing stimuli‐responsive magnets capable of chemo‐switching through mass transport. In most instances, research on low‐dimensional systems has concentrated on interlayer and interchain interactions. Unlike simple homogeneous layered magnets, this study demonstrates chemo‐switching in spin‐LMags and introduces a novel magnetic mechanism for phase transformations. In a layered antiferromagnet with a consistent backbone structure, when spins are symmetrically inserted between layers, a spin‐exchange frustration system can be envisioned because of the consistent AF nature of the interlayer interactions. By altering the relative strengths of the magnetic interactions *J*_LL_ and *J*_LS_ in the solvated and desolvated compounds, we successfully achieved reversible switching between the two spin‐frustrated phases, **FR‐e** and **FR‐h**, in a spin‐inserted [Ru_2_]_2_TCNQ‐based layered magnetic system. Note that the **FR‐h** phase of **2** in this study is visually a general F phase. In this study, the F phase of **2** can be regarded as the **FR‐h** phase because the *J*_LL_ of isostructural **1** is clearly AF. In **2‐DCE**, a frustrated phase **FR‐e** was detected under weak magnetic fields due to a delicate balance of interactions as |*J*_LS_| ≤ |*J*_LL_|. Conversely, in **2**, the interlayer interaction is AF (*J*_LL_ < 0); however, the ferromagnetic *J*_LS_ contribution surpasses the *J*_LL_ contribution (|*J*_LS_| >> |*J*_LL_|), concealing the FR phase and revealing a F phase. Thus, this F phase can be considered as a hidden frustrated state, **FR‐h**. This study highlights that the relative strengths of *J*_LL_ and *J*_LS_ are crucial. This is the first system to demonstrate a complete transition between **FR‐e** and **FR‐h** through guest adsorption/desorption, while maintaining the conditions for frustration. This research exemplifies a method for reversible magnetic phase conversion in low‐dimensional magnetic materials and confirms that insertion‐type spin hybrid magnets are promising candidates for creating magnetic switching devices. The findings of this study can contribute to future studies on controlling the adsorption of paramagnetic gases such as oxygen by controlling the internal magnetic field of a layered magnet.

## 4 Experimental Section

### General Procedures and Materials

Synthetic operations were conducted in an N_2_ atmosphere employing standard Schlenk‐line methods and a commercial glove box. Chemicals were of reagent grade and obtained from commercial sources. Solvents were dried with appropriate drying agents and distilled under nitrogen atmosphere. TCNQ and [MCp*_2_] were purchased from TCI and Sigma‐Aldrich Co., Ltd, respectively. [Ru_2_^II,II^(2,3,5‐F_3_ArCO_2_)_4_(THF)_2_]^[^ ^70^, ^71^ ^]^ was prepared following reported methods.

### Synthesis of [CoCp*_2_][{Ru_2_(2,3,5‐F_3_ArCO_2_)_4_}_2_(TCNQ)]•2DCE (**1‐DCE**)

Portions (2 mL) of a solution containing TCNQ (10.2 mg, 0.05 mmol) and [CoCp*_2_] (16.5 mg, 0.05 mmol) in DCM (100 mL) were poured into narrow‐diameter glass tubes (inner diameter of 8 mm). Subsequently, a mixed solvent of DCM/DCE in a volume ratio of 1:1 (1 mL) was added on the bottom layer as a buffer layer (middle layer). Finally, [Ru_2_^II,II^(2,3,5‐F_3_ArCO_2_)_4_(THF)_2_] (104.6 mg, 0.1 mmol) in DCE (100 mL) was separated into 2 mL portions and placed on the buffer layer of each tube (top layer). The tubes were sealed and left undisturbed for one month to allow the crystallization of block‐shaped black crystals of **1‐DCE** (yield: 9%). The crystallization solvents (2 molar amounts of DCE) in **1‐DCE** were easily eliminated when the fresh crystals were exposed to air, as proved by thermogravimetric analysis (Figure S7, Supporting Information), which made it difficult to accurately perform elemental analyses on the fresh samples. Therefore, the elemental analysis for the present compound was performed only for the solvent‐free sample **1**. Infrared (KBr), *ν* (C≡N), 2194, 2154 cm^−1^.

### Synthesis of [FeCp*_2_][{Ru_2_(2,3,5‐F_3_ArCO_2_)_4_}_2_(TCNQ)]•2DCE (**2‐DCE**)

The synthesis of the crystalline sample of **2‐DCE** for SCXRD analysis was analogous to that of **1‐DCE**, with the exception that [CoCp*_2_] was replaced with [FeCp*_2_] (yield: 22%). IR (KBr): *ν*_(C≡N)_ 2197 and 2156 cm^−1^.

### Preparation of Dried Samples [MCp*_2_][{Ru_2_(2,3,5‐F_3_ArCO_2_)_4_}_2_(TCNQ)] (**1**, M = Co; **2**, M = Fe)

Crystal samples of **1**/**2** were prepared by heating **1‐DCE**/**2‐DCE** at 353 K under vacuum for 12 h. Elemental analysis (%): calculated for C_68_H_50_CoF_24_N_4_O_16_Ru_4_, C (45.20), H (2.16), N (2.40). Found: C (44.80), H (2.26), N (2.47). Infrared (KBr) of **1**: ν (C≡N), 2196, 2155 cm^−1^. Elemental analysis (%): calculated for C_68_H_50_FeF_24_N_4_O_16_Ru_4_, C (45.25), H (2.15), N (2.39). Found: C (45.24), H (2.31), N (2.54). Infrared (KBr) of **2**: ν (C≡N), 2193, 2155 cm^−1^.

### Physical Characterization

IR spectra were recorded on a KBr pellet using a Jasco FT‐IR 4200 spectrophotometer. Thermogravimetric analyses were conducted on a Shimadzu DTG‐60H apparatus under a N_2_ atmosphere in the temperature range from room temperature to 400 °C at a heating rate of 5 °C min^−1^. PXRD patterns were collected using a Rigaku Ultima IV diffractometer supplying Cu Kα radiation (λ = 1.5418 Å). Magnetic susceptibility measurements were performed with a Quantum Design SQUID magnetometer (MPMS‐XL) in the temperature range of 1.8 to 300 K and under a dc magnetic field from –7 to 7 T. Polycrystalline samples embedded in liquid paraffin were analyzed. The experimental data were corrected for contributions from the sample holder, liquid paraffin, and diamagnetic effects calculated using Pascal constants.^[^ ^72^ ^]^

### Crystallography

Crystal data were collected using a CCD diffractometer (Rigaku Saturn724M+HyPix‐6000HE) with multilayer mirror monochromated Mo Kα radiation (λ = 0.71073 Å). Single crystals with dimensions of 0.095 × 0.119 × 0.147 mm, 0.051 × 0.115 × 0.185 mm, 0.065 × 0.09 × 0.145 mm, and 0.044 × 0.133 × 0.147 mm for **1‐DCE**, **2‐DCE**, **1**, and **2**, respectively, were mounted on a thin Kapton film using Nujol and cooled to 103 K in an N_2_ gas stream. Using Olex2,^[^ ^73^ ^]^ the crystal structures were solved with the SHELXT structure solution program using Intrinsic Phasing^[^ ^74^ ^]^ and refined with the SHELXL^[^ ^75^ ^]^ refinement package through least‐squares minimization. Anisotropic refinement of non‐hydrogen atoms was performed using the full‐matrix least‐squares method on *F* ^2^. Non‐hydrogen atoms were hydrogenated based on the theoretical background of structural chemistry, and hydrogen atoms were fixed in their positions on the carbon atoms. The crystallographic data, parameters for data collection, and specifics of the structure refinement are detailed in Table S1 (Supporting Information). These datasets have been deposited as CIFs at the Cambridge Data Centre as supplementary publication notes. CCDC‐ 2 448 178 for **1‐DCE**, 2 448 180 for **2‐DCE**, 2 448 179 for **1**, and 2 448 181 for **2**. Structural diagrams were generated using the Diamond software.^[^ ^76^ ^]^

[CCDC 2448178–2448181 contains the supplementary crystallographic data for this paper. These data can be obtained free of charge from The Cambridge Crystallographic Data Centre via www.ccdc.cam.ac.uk/data_request/cif.]

## Conflict of Interest

The authors declare no conflict of interest.

## Supporting information



Supporting Information

Supporting Information

## Data Availability

The data that support the findings of this study are available in the supplementary material of this article.
