# Supplementary material for: Guest‐Induced Reversible Phase Conversion via Spin Frustration Relief in Spin‐Intercalated Layered Antiferromagnets
Source: Adv Sci (Weinh). 2025 Jul 26;12(39):e07957. doi: 10.1002/advs.202507957 (PMC12533306; doi:10.1002/advs.202507957)
Supplement: Supplementary file 1 — Supporting Information [file ADVS-12-e07957-s001.pdf]

ADVANCED SCIENCE
Open Access

Supporting Information

for *Adv. Sci.*, DOI 10.1002/advs.202507957

Guest-Induced Reversible Phase Conversion via Spin Frustration Relief in Spin-Intercalated Layered Antiferromagnets

*Qingxin Liu, Honoka Nemoto, Wataru Kosaka and Hitoshi Miyasaka**

((Supporting Information can be included here using this template))

Supporting Information

Guest-Induced Reversible Phase Conversion via Spin Frustration Relief in Spin-Intercalated Layered Antiferromagnets

*Qingxin Liu, Honoka Nemoto, Wataru Kosaka, and Hitoshi Miyasaka**

Contents for SI

Contents for SI ········ S2

Table S1 Crystallographic data of **1-DCE** and **2-DCE** ········ S3

Table S2 Crystallographic data of **1** and **2** ········ S4

Table S3 The oxidation state of the [Ru_2] unit ········ S5

Table S4 Bond distances in the TCNQ moiety ········ S6

Table S5 M-Cp*$_{center}$ distances ········ S7

Fig. S1 Infrared spectra and Raman spectra ········ S8

Fig. S2 PXRD patterns ········ S9

Fig. S3 Structures of **2-DCE** ········ S10

Fig. S4 Structures of **1-DCE** and **1** ········ S11

Fig. S5 Packing views of **1-DCE** ········ S12

Fig. S6 Packing views of **2-DCE** ········ S13

Fig. S7 The TGA curves ········ S14

Fig. S8 Tilt angles in four compounds ········ S15

Fig. S9 χ-T and χT-T plots of fresh compounds at 1kOe ········ S16

Fig. S10 FCM, ZFCM and RM curves of **1-DCE** ········ S17

Relaxation behavior analysis of 1-DCE ········ S18

Fig. S11 Fitting result of **1-DCE** using the critical scaling model ········ S19

Fig. S12 dM/dH of **1-DCE** ········ S20

Field-induced ferromagnetism in 1-DCE and 1 ········ S21

Fig. S13 FCM, ZFCM and RM curves of **2-DCE** ········ S22

Fig. S14 Phase boundaries in **2-DCE** ········ S23

Spin-glassy behavior analysis of 2-DCE ········ S24

Fig. S15 Multiple peak fit results of χ'' of **2-DCE** with Gauss function ········ S25

Table S6 χ'' peak positions of fit peaks after separation ········ S26

Fig. S16 Fitting result of **2-DCE** using the critical scaling model ········ S27

Fig. S17 χ-T and χT-T plots of guest-free compounds at 1kOe ········ S28

Fig. S18 FCM, ZFCM and RM curves of **1** ········ S29

Fig. S19 dM/dH of **1** ········ S30

Fig. S20 FCM, ZFCM and RM curves of **2** ········ S31

Fig. S21 Proposed stepwise spin ordering processes of **2** ········ S32

Fig. S22 PXRD patterns of **1-DCE** and **1** in the de-/adsorption cycle ········ S33

References ········ S34

Table S1. Crystallographic data for **1-DCE** and **2-DCE**.

Compounds	**1-DCE**	**2-DCE**
Empirical formula	$C_{92}H_{58}Cl_4CoF_{24}N_4O_{16}Ru_4$	$C_{92}H_{58}Cl_4FeF_{24}N_4O_{16}Ru_4$
Formula weight	2536.43	2533.35
Temperature / K	103.15	103.15
Crystal system	monoclinic	monoclinic
Space group	*I*2/*a*	*I*2/*a*
a / Å	21.0756(7)	21.1037(5)
b / Å	20.9069(6)	20.8858(4)
c / Å	21.9844(5)	22.0044(4)
α / °	90	90
β / °	96.911(2)	97.480(2)
γ / °	90	90
Volume / Å^3	9616.5(5)	9616.3(3)
Z	4	4
ρ_{calc} / g·cm^{-3}	1.752	1.750
μ / mm^{-1}	1.007	0.985
F_{000}	5012.0	5008.0
Crystal size / mm^3	0.147 × 0.119 × 0.095	0.185 × 0.115 × 0.051
Radiation	Mo *K*α (λ = 0.71073)	Mo *K*α (λ = 0.71073)
2θ range for data collection / °	5.06 to 54.998	4.432 to 50.696
Index ranges	$-25 \leq h \leq 26, -27 \leq k \leq 22, -28 \leq l \leq 28$	$-25 \leq h \leq 25, -25 \leq k \leq 24, -19 \leq l \leq 26$
Reflections collected	38898	32806
Independent reflections	10809 [R_{int} = 0.0355, R_{sigma} = 0.0339]	8772 [R_{int} = 0.0218, R_{sigma} = 0.0142]
Data/restraints/parameters	10809/1411/962	8772/1040/953
Goodness-of-fit on F^2	1.025	1.087
Final R^{1-2} indexes [I>=2σ (I)]	R_1 = 0.0637, wR_2 = 0.1677	R_1 = 0.0844, wR_2 = 0.2410
Final R^2 indexes [all data]	R_1 = 0.0818, wR_2 = 0.1804	R_1 = 0.0897, wR_2 = 0.2503
CCDC No.	2448178	2448180

$R_1 = \sum||F_c| - |F_o|| / \sum|F_o|; wR_2 = [\sum w(F_o^2 - F_c^2)^2/\sum w(F_o^2)^2]^{1/2}$

Table S2. Crystallographic data for **1** and **2**.

Compounds	**1**	**2**
Empirical formula	$C_{88}H_{50}CoF_{24}N_4O_{16}Ru_4$	$C_{88}H_{50}FeF_{24}N_4O_{16}Ru_4$
Formula weight	2338.52	2335.45
Temperature / K	103.15	103.15
Crystal system	monoclinic	monoclinic
Space group	$P2_1/c$	$P2_1/c$
a / Å	10.4370(3)	10.4919(3)
b / Å	19.8777(6)	20.0403(7)
c / Å	21.5127(7)	21.6972(8)
α / °	90	90
β / °	98.246(3)	99.154(3)
γ / °	90	90
Volume / Å^3	4417.0(2)	4504.0(3)
Z	2	2
ρ_{calc} / g·cm^{-3}	1.758	1.722
μ / mm^{-1}	0.971	0.929
F_{000}	2306.0	2304.0
Crystal size / mm^3	0.145 × 0.09 × 0.065	0.147 × 0.133 × 0.044
Radiation	Mo $K\alpha$ (λ = 0.71073)	Mo $K\alpha$ (λ = 0.71073)
2θ for data collection / °	5.058 to 54.996	5.018 to 55.992
Index ranges	$-12 \leq h \leq 13, -25 \leq k \leq 25, -19 \leq l \leq 27$	$-13 \leq h \leq 11, -26 \leq k \leq 26, -28 \leq l \leq 25$
Reflections collected	35387	37126
Independent reflections	10104 [R_{int} = 0.0327, R_{sigma} = 0.0343]	10755 [R_{int} = 0.0418, R_{sigma} = 0.0449]
Data/restraints/parameters	10104/0/664	10755/0/664
Goodness-of-fit on F^2	1.159	1.167
Final R^{1-2} indexes [I>=2σ (I)]	R_1 = 0.0622, wR_2 = 0.1442	R_1 = 0.0641, wR_2 = 0.1398
Final R^2 indexes [all data]	R_1 = 0.0791, wR_2 = 0.1511	R_1 = 0.0901, wR_2 = 0.1500
CCDC No.	2448179	2448181

$R_1 = \sum||F_c| - |F_o|| / \sum|F_o|; wR_2 = [\sum w(F_o^2 - F_c^2)^2/\sum w(F_o^2)^2]^{1/2}$

Table S3. Selected bond lengths (Å) for **1-DCE**, **2-DCE**, **1** and **2**.

Compound	Asymmetric $[Ru_2]$ unit		Ru-O band / Å<br>Ru1/1A-O1 (Ru2/2A-O2)	Ru1/1A-O3 (Ru2/2A-O4)	Ru1/1A-O5 (Ru2/2A-O6)	Ru1/1A-O7 (Ru2/2A-O8)	average	Charge
**1-DCE**	Part-A	Ru1	2.030(4)	2.091(4)	2.108(4)	2.047(4)	2.069(2)	II,II
		Ru2	2.035(4)	2.089(4)	2.103(4)	2.056(4)	2.071(2)	
	Part-B	Ru1A	2.309(8)	2.005(9)	1.853(8)	2.172(9)	2.085(4)	II,II
		Ru2A	2.307(8)	1.987(9)	1.847(8)	2.189(9)	2.082(4)	
**2-DCE**	Part-A	Ru1	2.097(4)	2.061(5)	2.036(4)	1.068(4)	2.066(2)	II,II
		Ru2	2.031(4)	2.076(5)	2.104(4)	2.049(5)	2.066(2)	
	Part-B	Ru1A	1.804(10)	2.191(11)	2.353(10)	1.99(1)	2.080(5)	II,II
		Ru2A	2.135(6)	2.020(6)	2.001(6)	2.108(6)	2.066(3)	
**1**		Ru1	2.046(4)	2.074(4)	2.053(4)	2.055(4)	2.057(2)	II,II
		Ru2	2.066(4)	2.048(4)	2.063(4)	2.056(4)	2.058(2)	II,II
**2**		Ru1	2.058(3)	2.083(4)	2.061(4)	2.065(4)	2.065(2)	II,II
		Ru2	2.080(4)	2.058(4)	2.065(4)	2.065(4)	2.067(2)	II,II

The oxidation state of $[Ru_2]$ unit can be known from the Ru–O_{eq} length (O_{eq} = equatorial oxygen atoms), which is quite sensitive to the oxidation state of the $[Ru_2]$ unit and to be 2.06–2.07 Å for $[Ru_2^{II,II}]$ and 2.02–2.03 Å for $[Ru_2^{II,III}]^+$.[1],[2] The average Ru–O_{eq} lengths of different disorder parts in four compounds almost are in the range of 2.06–2.08 Å, indicating that all the $[Ru_2]$ units are $[Ru_2^{II,II}]$.

Table S4. Bond distances in the TCNQ moiety and degree of charge transfer (ρ) estimated from the Kistenmacher relationship.[3]

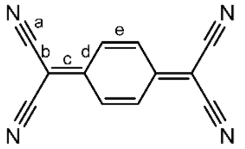

Compound	charge	*a*	*b*	*c*	*d*	*e*	ρ
TCNQ[4]	0	1.140(1)	1.441(1)	1.374(3)	1.448(4)	1.346(3)	0 (fix)
RbTCNQ[5]	−1	1.153(7)	1.416(8)	1.420(1)	1.423(3)	1.373(1)	−1 (fix)
		1.145(6)	1.410(6)	1.427(6)	1.417(6)	1.361(6)	
		1.135(6)	1.408(6)		1.421(6)		
**1-DCE**		Av. 1.140	Av.	Av.	Av.	Av.	−1.19(10)
		(4)	1.409(4)	1.427(6)	1.419(4)	1.361(6)	
		1.144(7)	1.401(7)	1.437(8)	1.407(7)	1.364(8)	
		1.153(7)	1.406(7)		1.408(7)		
**2-DCE**		Av	Av	Av	Av	Av	−1.47(13)
		1.148(5)	1.404(5)	1.437(8)	1.408(5)	1.364(8)	
		1.135(7)	1.403(8)	1.421(7)	1.417(7)	1.350(7)	
		1.131(7)	1.407(8)		1.410(7)		
**1**		Av.	Av.	Av.	Av.	Av.	−1.21(12)
		1.133(5)	1.405(6)	1.421(7)	1.408(5)	1.350(7)	
		1.155(7)	1.406(7)	1.432(7)	1.423(7)	1.360(7)	
		1.145(7)	1.408(7)		1.406(7)		
**2**		Av.	Av.	Av.	Av.	Av.	−1.31(12)
		1.150(5)	1.407(5)	1.432(7)	1.414(5)	1.360(7)	

The charge state of TCNQ can be roughly estimated from the local bond lengths of TCNQ using the Kistenmacher relationship $\rho = A[c/(b + d)] + B$, where b, c, and d are the respective bond lengths for the 7,9-, 1,7-, and 1,2-positioned C–C pairs in the TCNQ moiety; parameters $A = -41.667$ and $B = 19.833$, evaluated from $TCNQ^0$ ($\rho = 0$) and Rb^+TCNQ^- ($\rho = -1$).[4],[5],[6] The calculated ρ values are −1.19(10), −1.47(13), −1.21(12) and −1.31(12) Å for **1-DCE**, **2-DCE**, **1** and **2**, respectively, indicating the monoanion radical form of TCNQ.

Table S5. M-Cp*$_{center}$ distances and the electronic states of intercalated [MCp*$_2$] in **1-DCE**, **2-DCE**, **1**, and **2**.

Compounds	Metal of [MCp*$_2$]	M-Cp*$_{center}$ / Å	Electronic state
**1-DCE**	Co1 in Part-A<br>Co1A in Part-B	1.6483(1)<br>1.5858(2)	[Co^{III}Cp*$_2$]$^+$
**2-DCE**	Fe1 in Part-A<br>Fe1A in Part-B	1.6987(1)<br>1.7183(8)	[Fe^{III}Cp*$_2$]$^+$
**1**	Co1	1.6340(36)	[Co^{III}Cp*$_2$]$^+$
**2**	Fe1	1.6994(30)	[Fe^{III}Cp*$_2$]$^+$

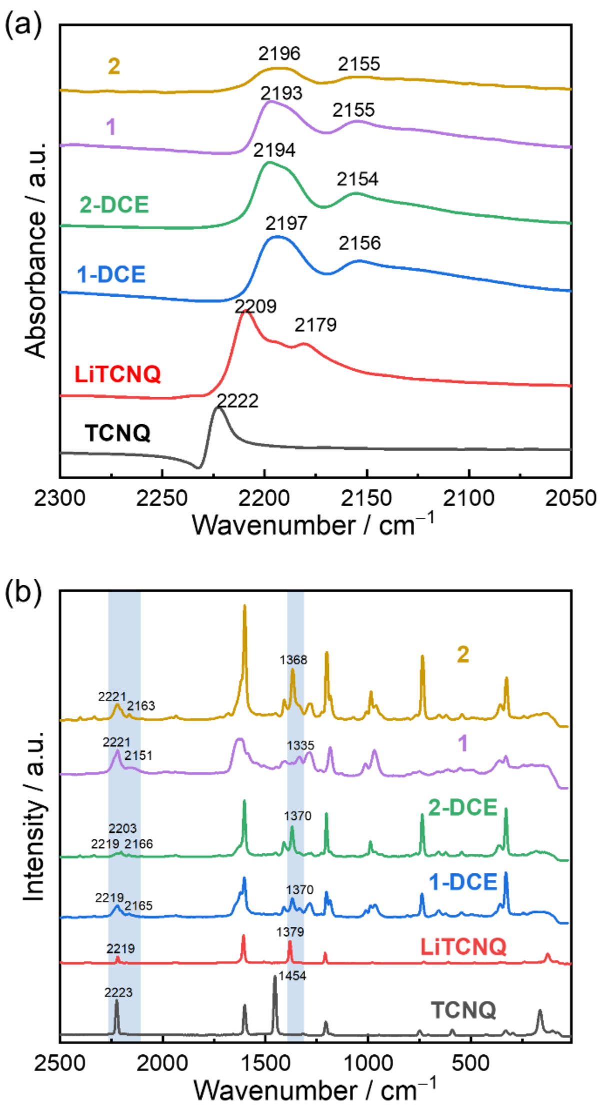

Figure S1. Infrared spectra (a) and Raman spectra (b) of **1-DCE**, **2-DCE**, **1**, **2**, TCNQ, and LiTCNQ measured at room temperature.

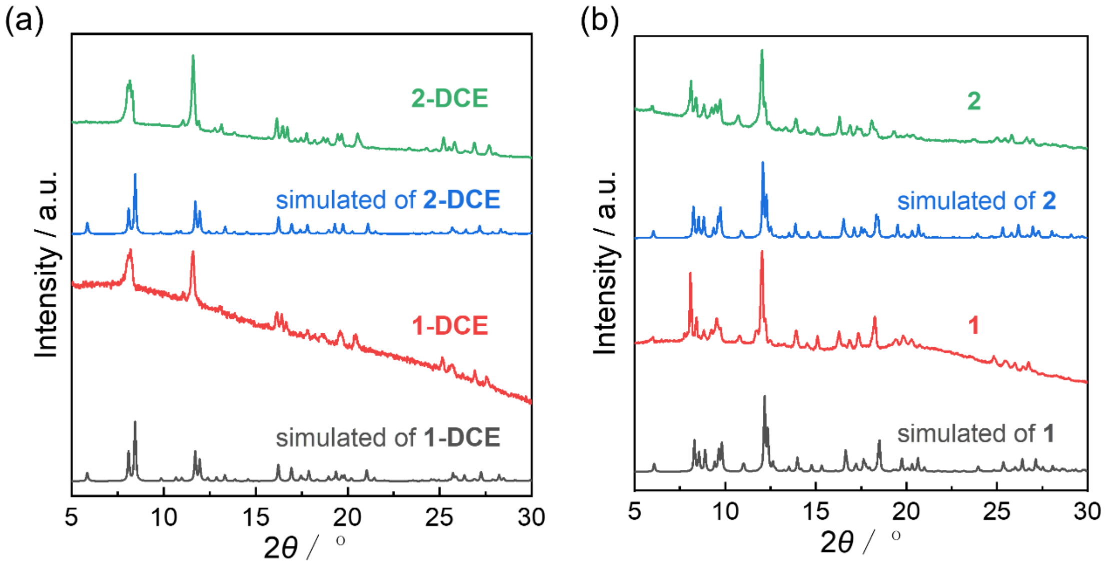

Figure S2. PXRD patterns of solvated (a) and desolvated (b) compounds at room temperature. The simulated PXRD patterns are obtained based on the SCXRD data.

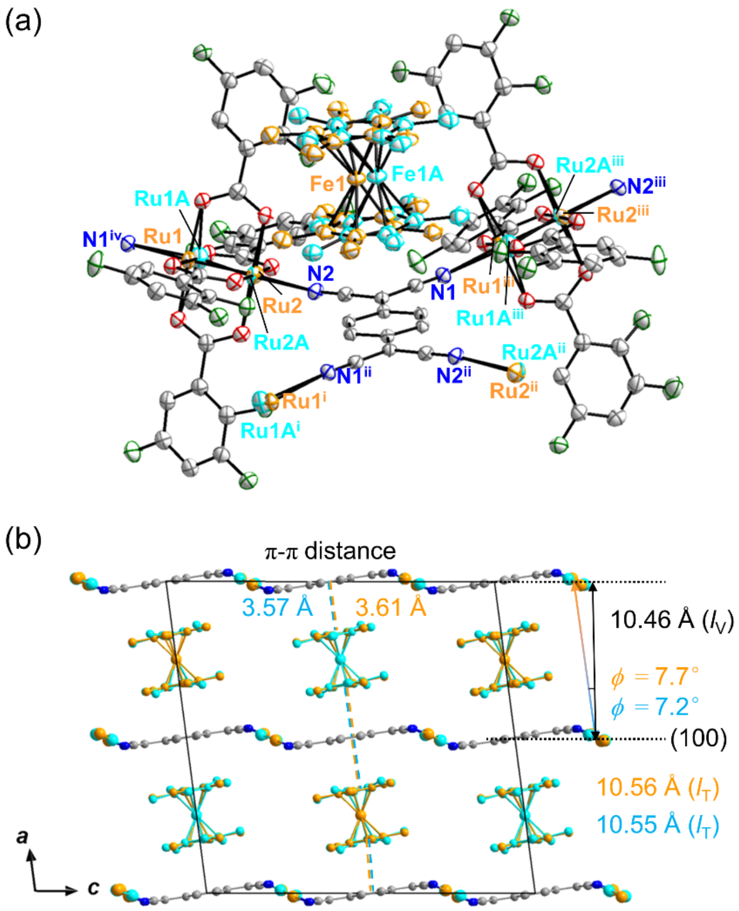

Figure S3. Structures of solvated **2-DCE**. (a) ORTEP representation of the formula unit and (b) packing view along the *b*-axis for **2-DCE**, where the crystallization of DCE, hydrogen atoms, and 2,3,5-$F_3ArCO_2^-$ ligands (only in b) around the Ru centers are omitted for clarity. The disordered Part-A and Part-B are colored orange and bright blue, respectively; the remaining ordered O, C, N, and F atoms are represented in red, gray, blue, and green, respectively. The symmetry operations are (i) $x, -y + 3/2, z + 1/2$, (ii) $-x + 1, -y + 1, -z + 2$, (iii) $-x + 1, y - 1/2, -z + 3/2$, and (iv) $-x + 1, y + 1/2, -z + 3/2$.

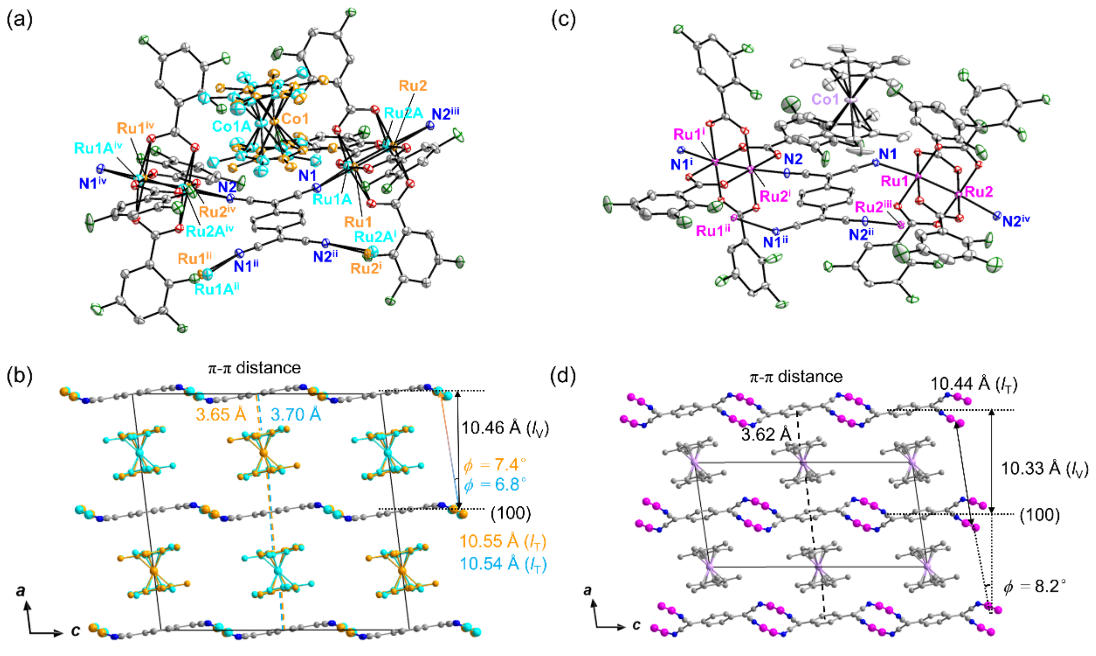

Figure S4. Structures of solvated **1-DCE** and the desolvated **1**. (a) ORTEP representation of the formula unit and (b) packing view along the *b*-axis for **1-DCE**, where crystallization DCE, hydrogen atoms and 2,3,5-$F_3ArCO_2^-$ ligands (only in b) around the Ru centers are omitted for clarity. The disordered Part-A and Part-B are colored in orange and bright blue, respectively; the remaining ordered O, C, N, and F atoms are represented in red, gray, blue, and green, respectively. The symmetry operations are (i) $x, -y + 1/2, z - 1/2$, (ii) $-x + 1, -y + 1, -z + 1$, (iii) $-x + 1, y - 1/2, -z + 3/2$, and (iv) $-x + 1, y + 1/2, -z + 3/2$. (c) ORTEP representation of the formula unit and (d) packing view along the *b*-axis for **1**, where crystallization DCE, hydrogen atoms and 2,3,5-$F_3ArCO_2^-$ ligands (only in d) around the Ru centers are omitted for clarity. The O, C, N, F, Co, and Ru atoms are represented in red, gray, blue, green, lavender, and purple, respectively. The symmetry operations are (i) $-x + 1, y - 1/2, -z + 1/2$, (ii) $-x + 1, -y + 1, -z + 1$, (iii) $x, -y + 3/2, z + 1/2$, and (iv) $-x + 1, y + 1/2, -z + 1/2$.

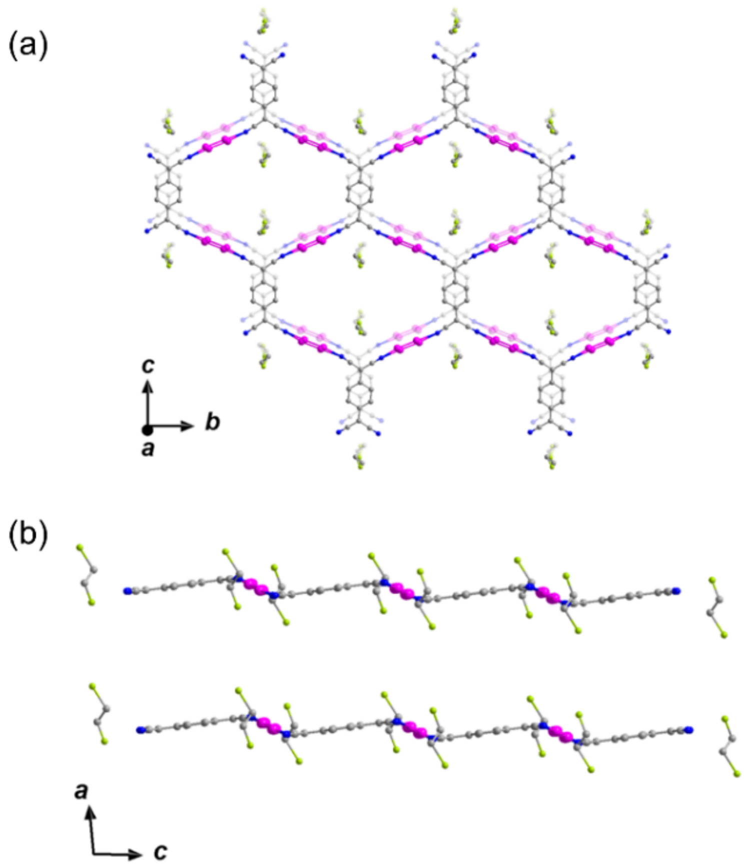

Figure S5. Packing views of **1-DCE** along the (100) plane (a) and *b*-axis (b). DCE molecules are located two sides of TCNQ moieties. The hydrogen atoms and 2,3,5-$F_3ArCO_2^-$ ligands and [$CoCp^*_2$] molecules are omitted for clarity. The O, C, N, Cl and Ru atoms are represented in red, gray, blue, lime, and purple, respectively.

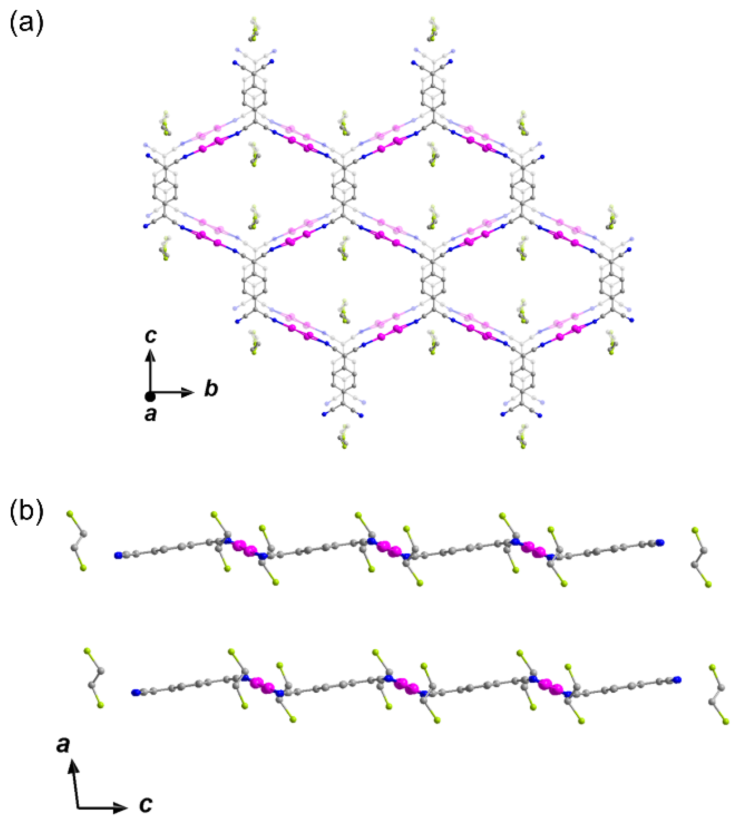

Figure S6. Packing views of **2-DCE** along the (100) plane (a) and *b*-axis (b). DCE molecules are located two sides of TCNQ moieties. The hydrogen atoms and 2,3,5-$F_3ArCO_2^-$ ligands and [FeCp*$_2$] molecules are omitted for clarity. The O, C, N, Cl and Ru atoms are represented in red, gray, blue, lime, and purple, respectively.

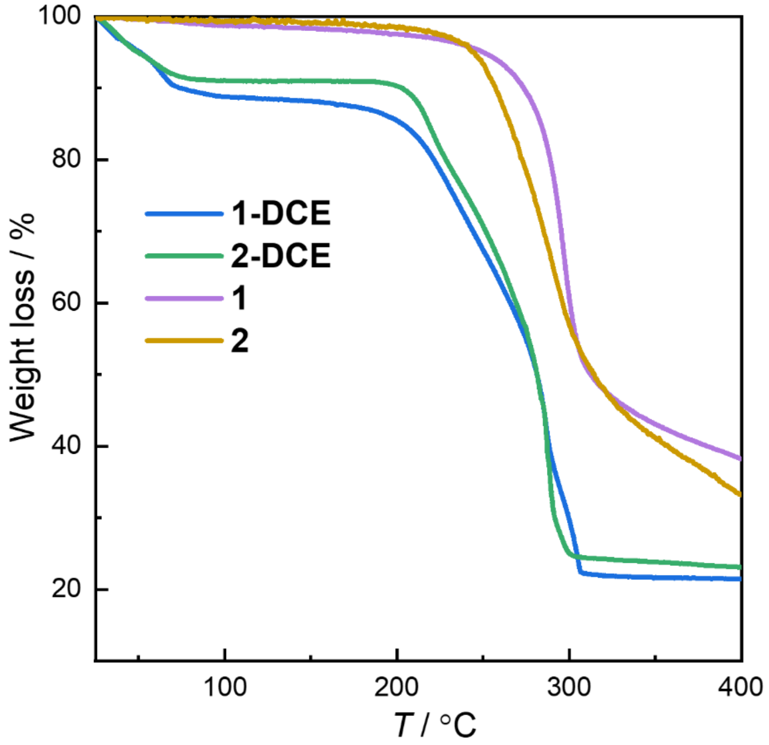

Figure S7. The TGA curves of **1-DCE**, **2-DCE**, **1**, and **2** with a heating rate of 5 °C/min^{-1} under N_2 flow.

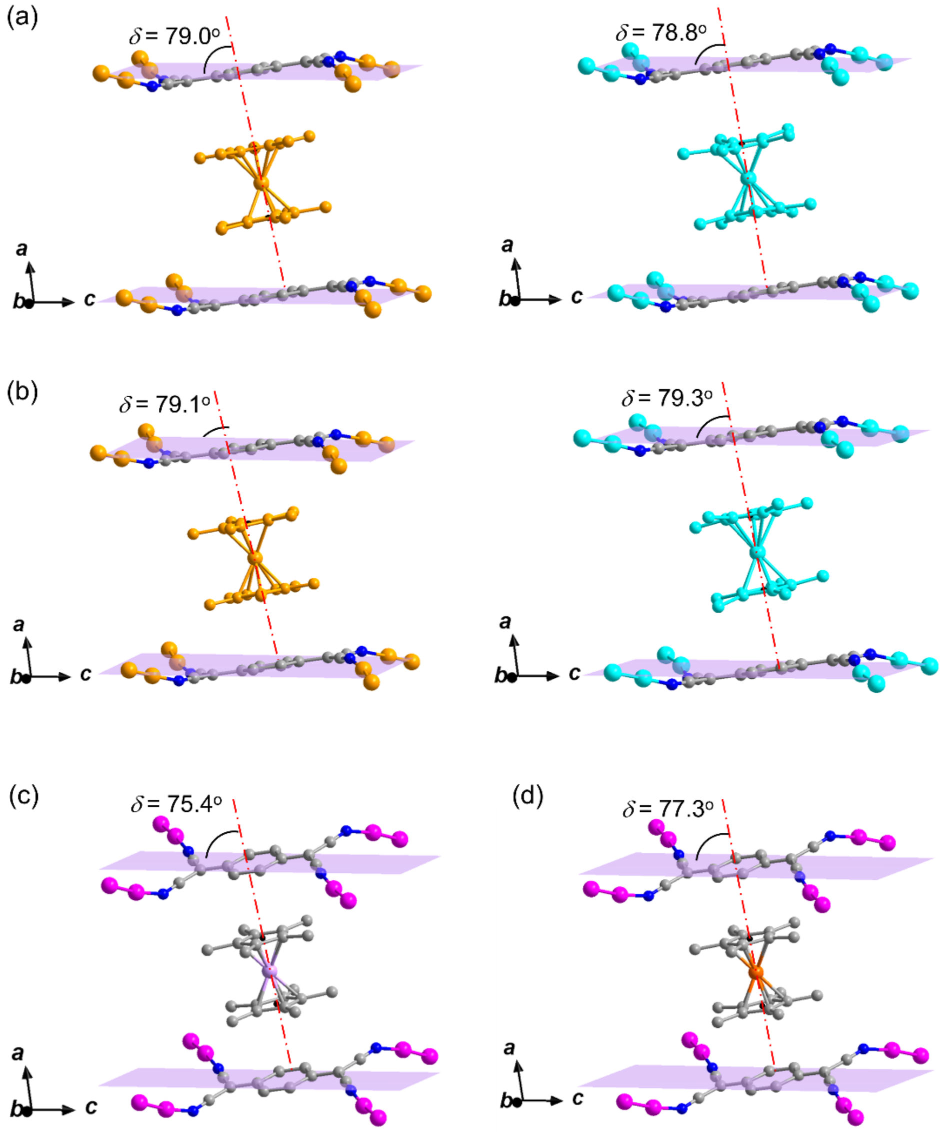

Figure S8. The tilt angels (δ) between the main axis of [MCp*$_2$] and D_2A layer (violet planes) in compounds **1-DCE** (a), **2-DCE** (b), **1** (c), and **2** (d). The red dotted lines represent the main axis of [MCp*$_2$], which are defined by the axis passing through centers of Cp* rings. The disordered part-A and part-B in **1-DCE** and **2-DCE** are colored in orange and bright blue, respectively; the remaining ordered O, C, N, F, Ru, Co, and Fe atoms are represented in red, gray, blue, green, purple, lavender, and orange, respectively.

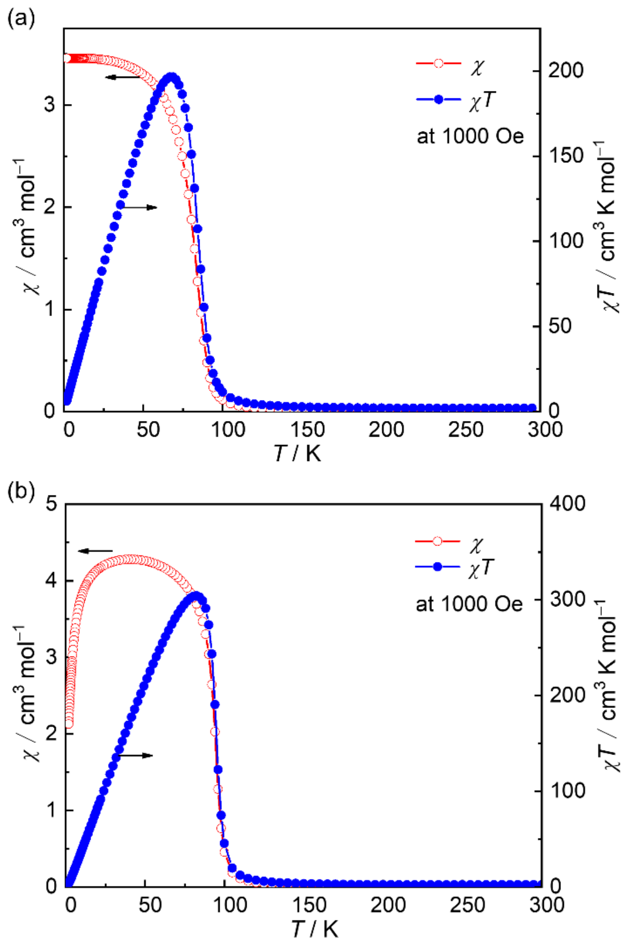

Figure S9. Temperature dependence of χ and χT of **1-DCE** (a) and **2-DCE** (b) measured at 1 kOe.

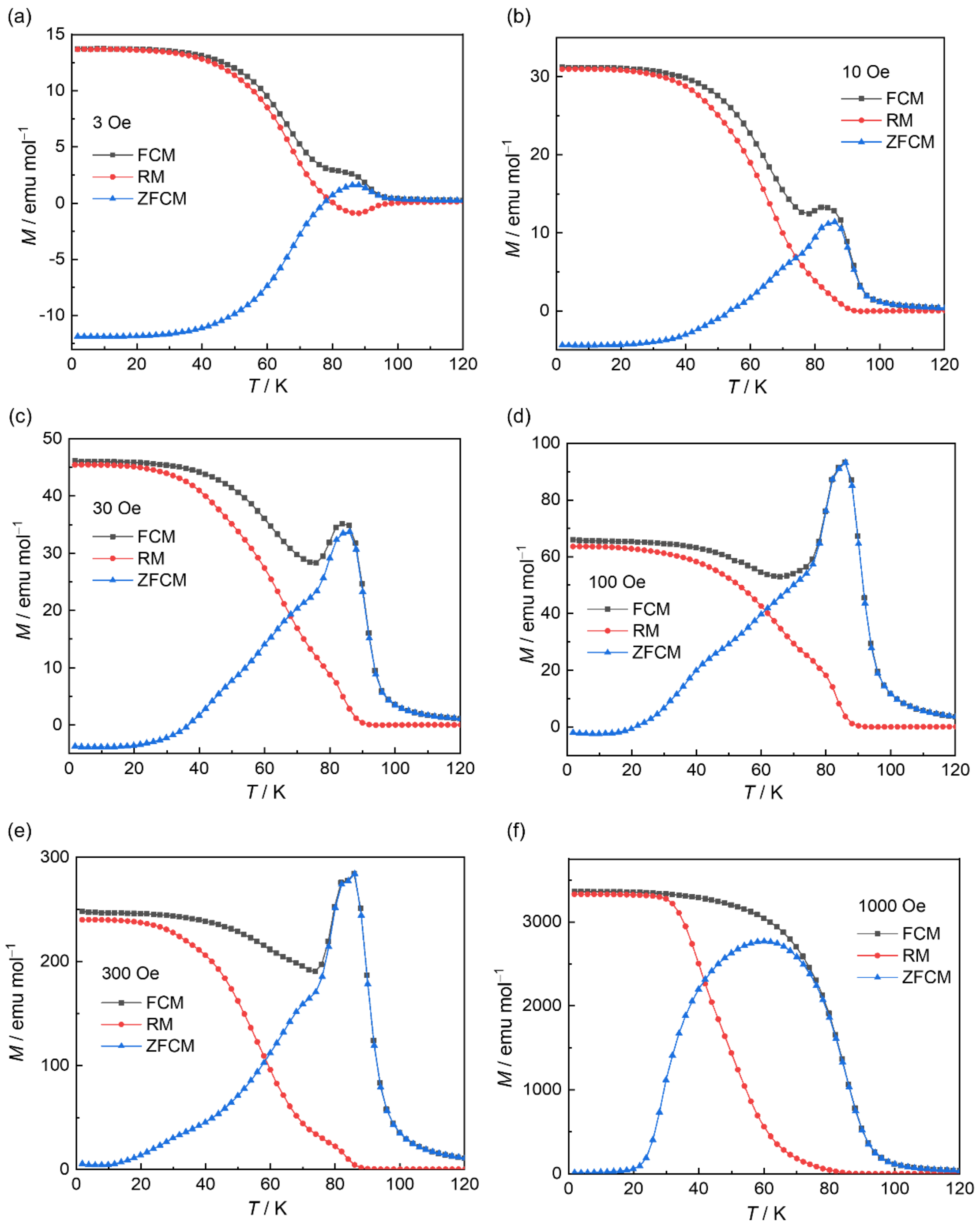

Figure S10. Field-cooled magnetization (FCM), zero-field-cooled magnetization (ZFCM), and remnant magnetization (RM) curves of **1-DCE** measured from 120 K to 1.8 K under different dc fields.

Relaxation behavior in 1-DCE using the critical scaling model.

As shown in Figure 2b, χ'' below 80 K of **1-DCE** shows an obvious frequency-dependent relaxation phenomenon. In the spin glass systems, the frequency-dependent relaxation follows the critical scaling model: $\tau = \tau_0 \cdot [\frac{T_B}{T_{SG}} - 1]^{-zv}$, where τ_0 is the single spin-flip relaxation time, T_{SG} is the spin glass temperature when f is equal to zero, zv is the dynamic critical exponent and T_B was defined as the value at the maximum point of relaxation peaks in $\chi''-T$ plot. For the canonical spin glass systems, the parameters of τ_0 and zv are in the range of 10^{-12}–10^{-13} and 4−12, respectively.[7],[8],[9] To investigate the relaxation behavior in **1-DCE**, dynamic slowing analysis was evaluated using the critical scaling model.

The best fitting result gives reasonable parameters (Figure S11), that is, τ_0, zv and T_{SG} are 5.0×10^{-6} s, 12.4, and 42.6 K, respectively. These parameters indicate the frequency-dependent χ'' in **1-DCE** is not spin-glassy characterized relaxation.

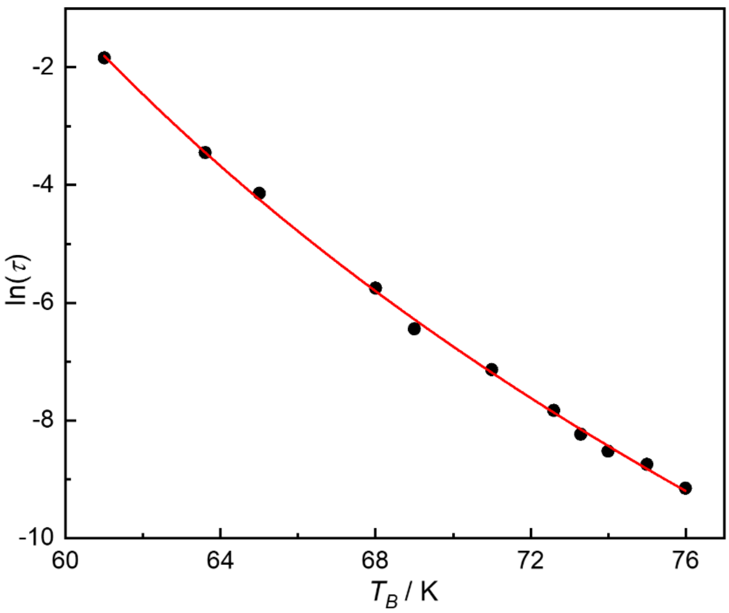

Figure S11. Plot of ln(τ) *versus* T_B for frequency-dependent behavior of **1-DCE**, in which the red line represents the non-linear fitting using the critical scaling approach.

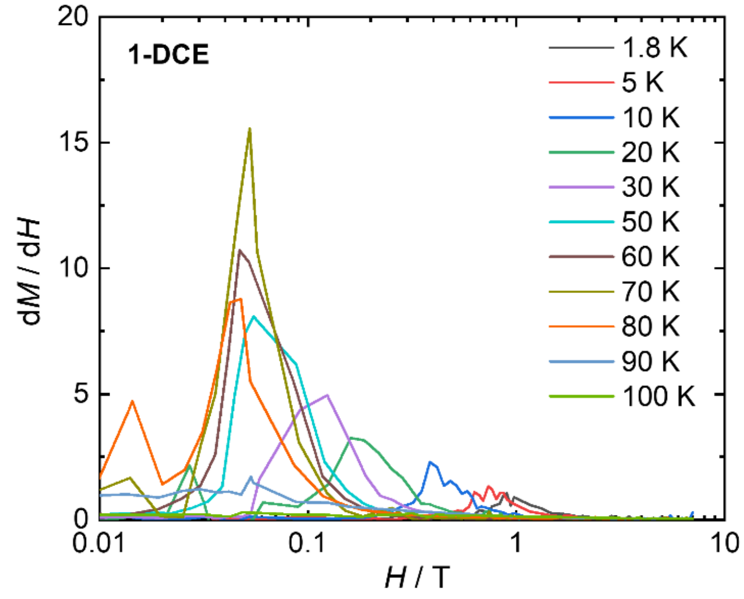

Figure S12. d*M*/d*H* of **1-DCE** for the initial field sweep from 0 to 7 T at different temperatures.

Field-induced ferromagnetism in 1-DCE and 1.

Generally, antiferromagnets do not exhibit remanent magnetization (M_r) or coercivity (H_c). However, **1-DCE** and **1** showed M_r and H_c, even though they have an antiferromagnetically ordered ground state. This is due to a unique characteristic to [{$Ru_2(RCO_2)_4$}$_2$TCNQR_x]-based layered antiferromagnet, in which once the compound is converted to a ferromagnetic arrangement by applying a high magnetic field, it does not revert to the original antiferromagnetic phase when the magnetic field is removed, but maintains the ferromagnetic arrangement; this property is called "field-induced ferromagnetism". By raising the temperature above T_N and then cooling under a weak magnetic field, the compound reverts to the antiferromagnetic phase. The origin of the magnetic field-induced ferromagnetism is unclear, but it is probably due to the strong magnetic anisotropy of [Ru_2] and the anisotropy of the layered structure.

So far, this phenomenon has always been observed in our layered [{$Ru_2(RCO_2)_4$}$_2$TCNQR_x] systems with antiferromagnetic order as the ground state.[10],[11],[12],[13]

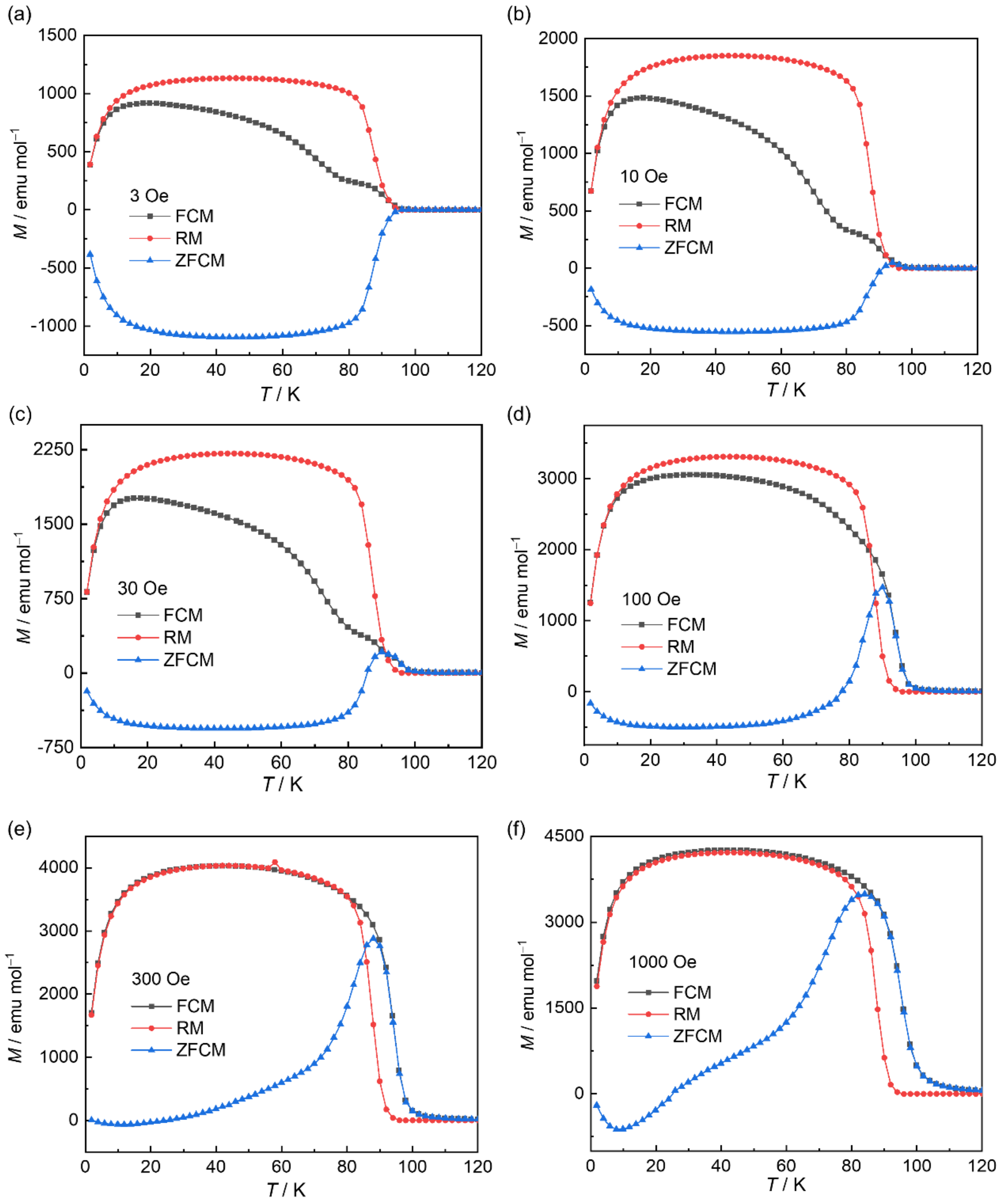

Figure S13. Field-cooled magnetization (FCM), zero-field-cooled magnetization (ZFCM), and remnant magnetization (RM) curves of **2-DCE** measured from 120 K to 1.8 K under different dc fields.

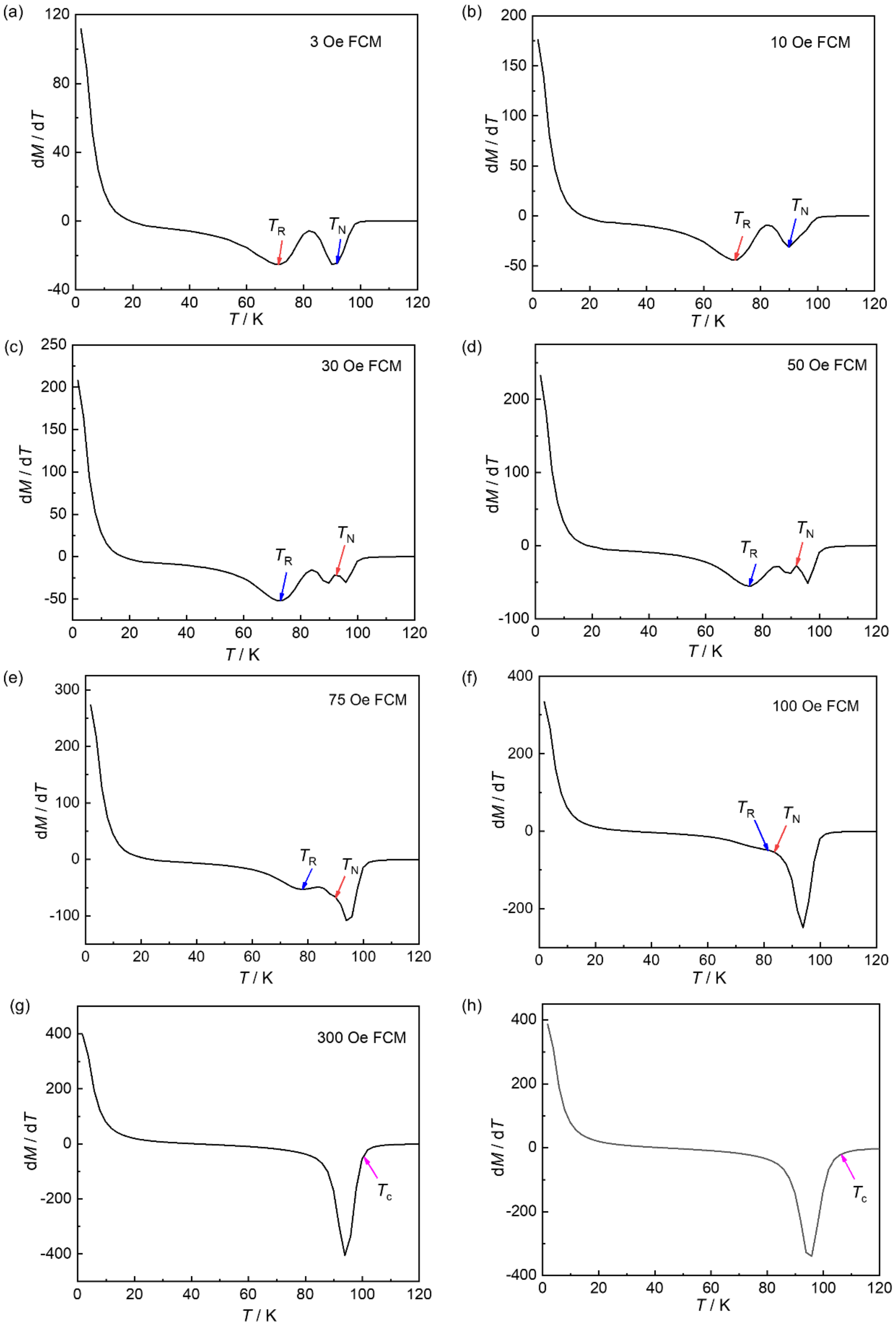

Figure S14. Phase boundaries in **2-DCE**: T_R, T_N and T_c, defined by the d*M*/d*T* based on the FCM curves at several dc fields.

Spin-glassy behavior in 2-DCE using the critical scaling model.

As shown in Figure 3b, both χ' and χ'' below 80 K of **2-DCE** show obvious frequency-dependent relaxation phenomenon. It was assigned to be spin-glassy behavior based on the shift parameter (φ = 0.0082). To confirm the relaxation behavior in **2-DCE**, dynamic slowing analysis was evaluated using the critical scaling model, showing as below:

$\tau = \tau_0 \cdot [\frac{T_B}{T_{SG}} - 1]^{-zv}$ (τ_0 is the single spin-flip relaxation time, T_{SG} is the spin glass temperature when f is equal to zero, zv is the dynamic critical exponent and T_B was defined as the value at the maximum point of relaxation peaks in $\chi''-T$ plot after multiple peak fit with Gauss function in Origin Pro2018.[14] The peak separation results are shown in Figure S15 and Table S6. Here, positions of fit peak 2 were almost unchanged, while fit peak 1 shifted to higher temperature with increasing frequency. The best fitting result gives reasonable parameters (Figure S16), that is, τ_0, zv and T_{SG} are 1.1×10^{-12} s, 7.3, and 73.6 K, respectively. From this analysis, the value of zv is in the typical range of a spin glass system between 4 and 12.[7] The τ_0 order of magnitude also matches with the canonical spin glass (10^{-12}–10^{-13}).[8],[9] Consequently, the relaxation behavior below 80 K in ac susceptibility should be ascribed to spin-glassy characterized relaxation.

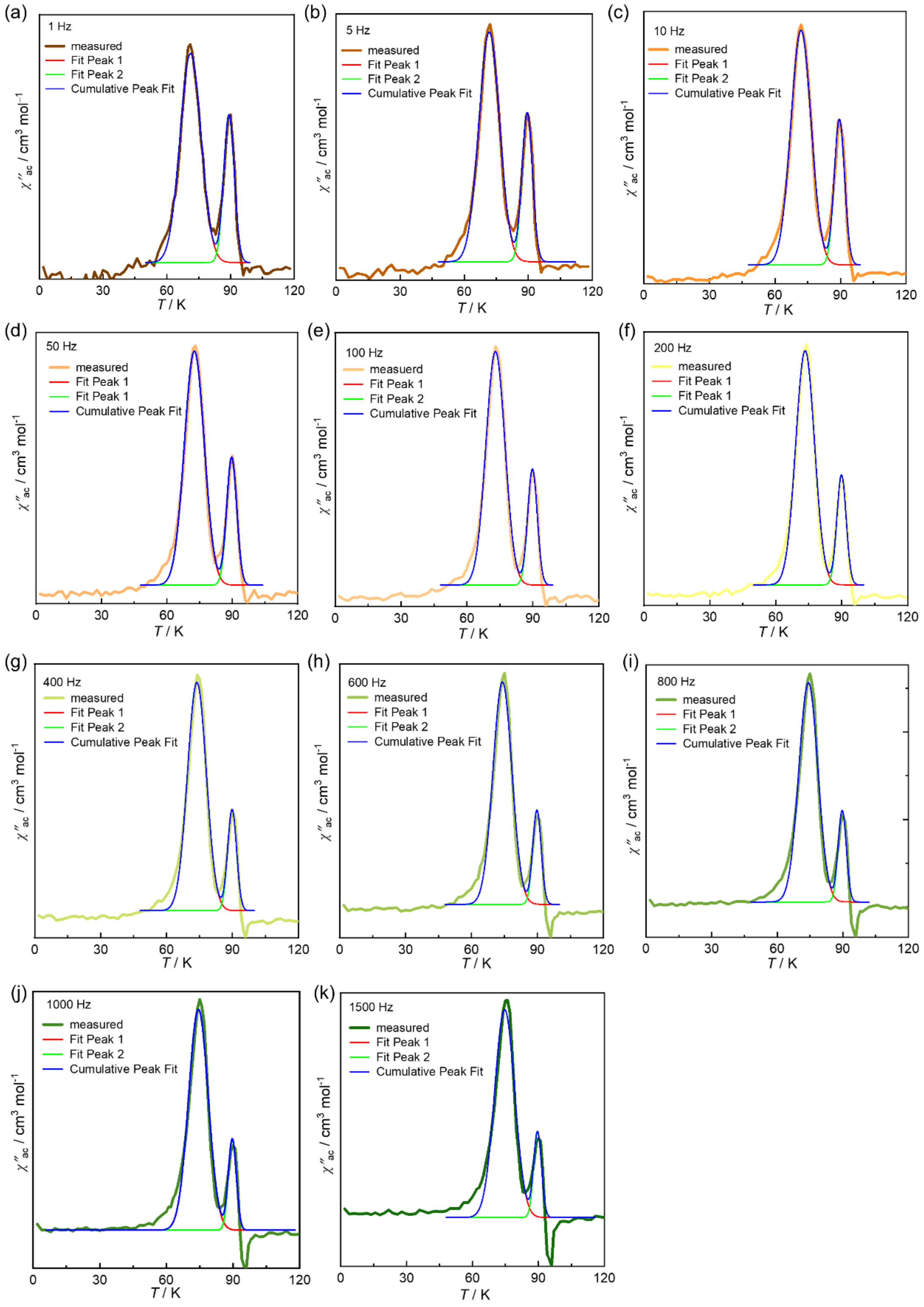

Figure S15. Multiple peak fit results of χ" at different frequency of **2-DCE** with Gauss function using Origin Pro 2018.

Table S6. χ'' peak positions of fit peaks of **2-DCE** after separation with Gauss function.

Frequency / Hz	Position of fit peak 1 / K	Position of fit peak 2 / K
1	71.15(10)	89.25(10)
5	71.66(9)	89.43(10)
10	71.94(10)	89.50(11)
50	72.58(11)	89.72(14)
100	72.95(11)	89.79(16)
200	73.36(11)	89.91(17)
400	73.81(13)	89.8 (2)
600	74.08(14)	89.8 (2)
800	74.26(15)	89.8(2)
1000	74.40(13)	89.7(2)
1500	74.62(19)	89.6(3)

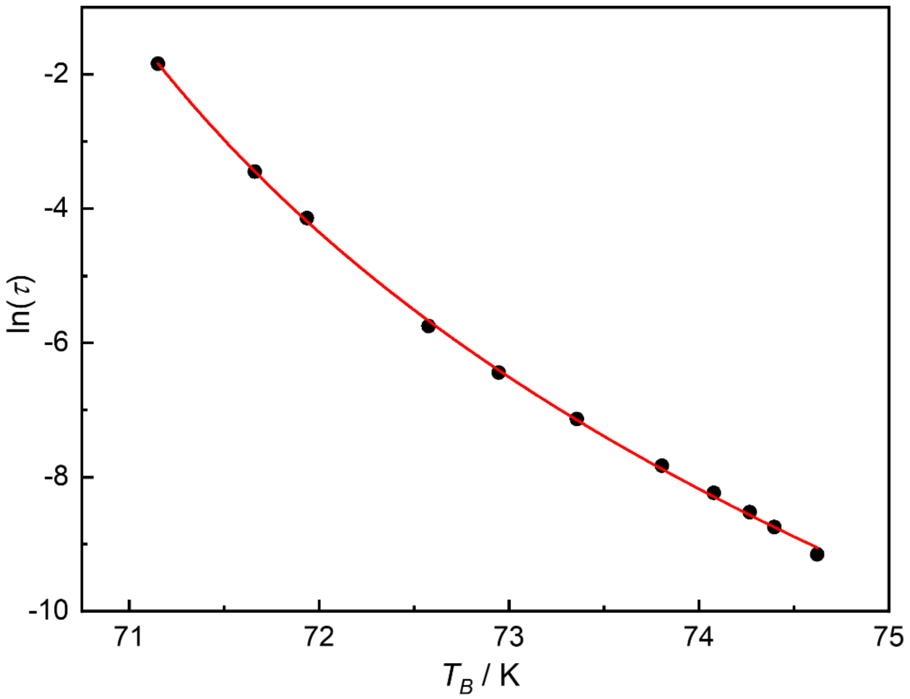

Figure S16. Plot of ln(τ) *versus* T_B (positions of peak 1) for frequency-dependent behavior of **2-DCE**, in which the red line represents the non-linear fitting using the critical scaling approach.

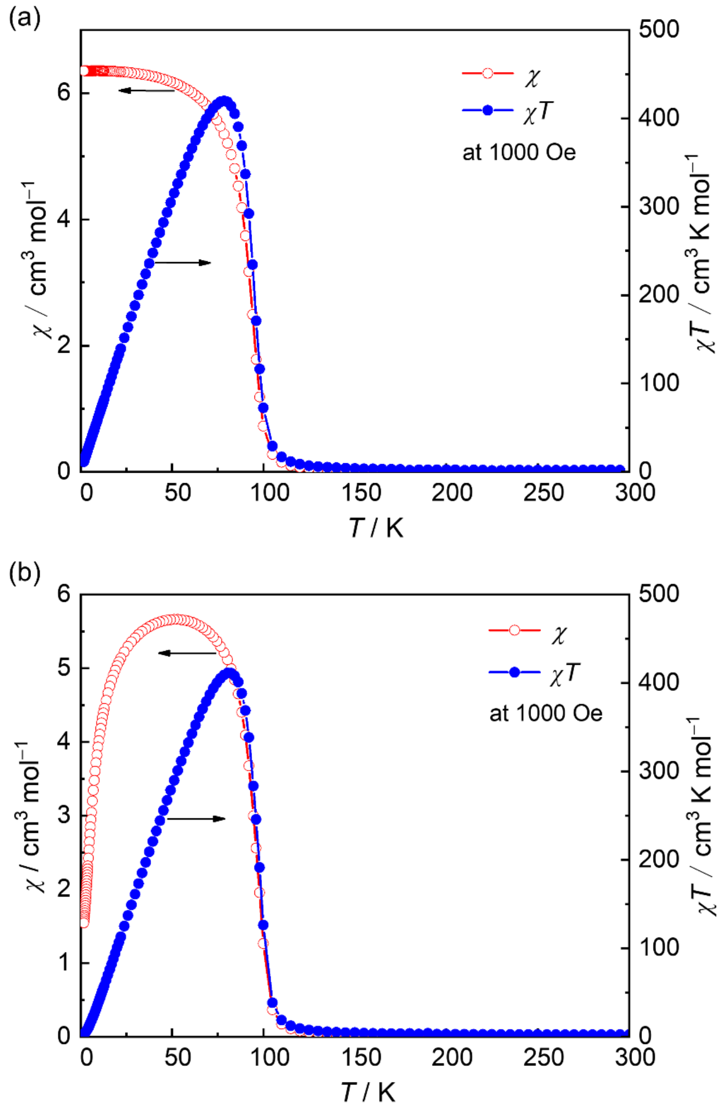

Figure S17. Temperature dependence of χ and χT of **1** (a) and **2** (b) measured at 1 kOe.

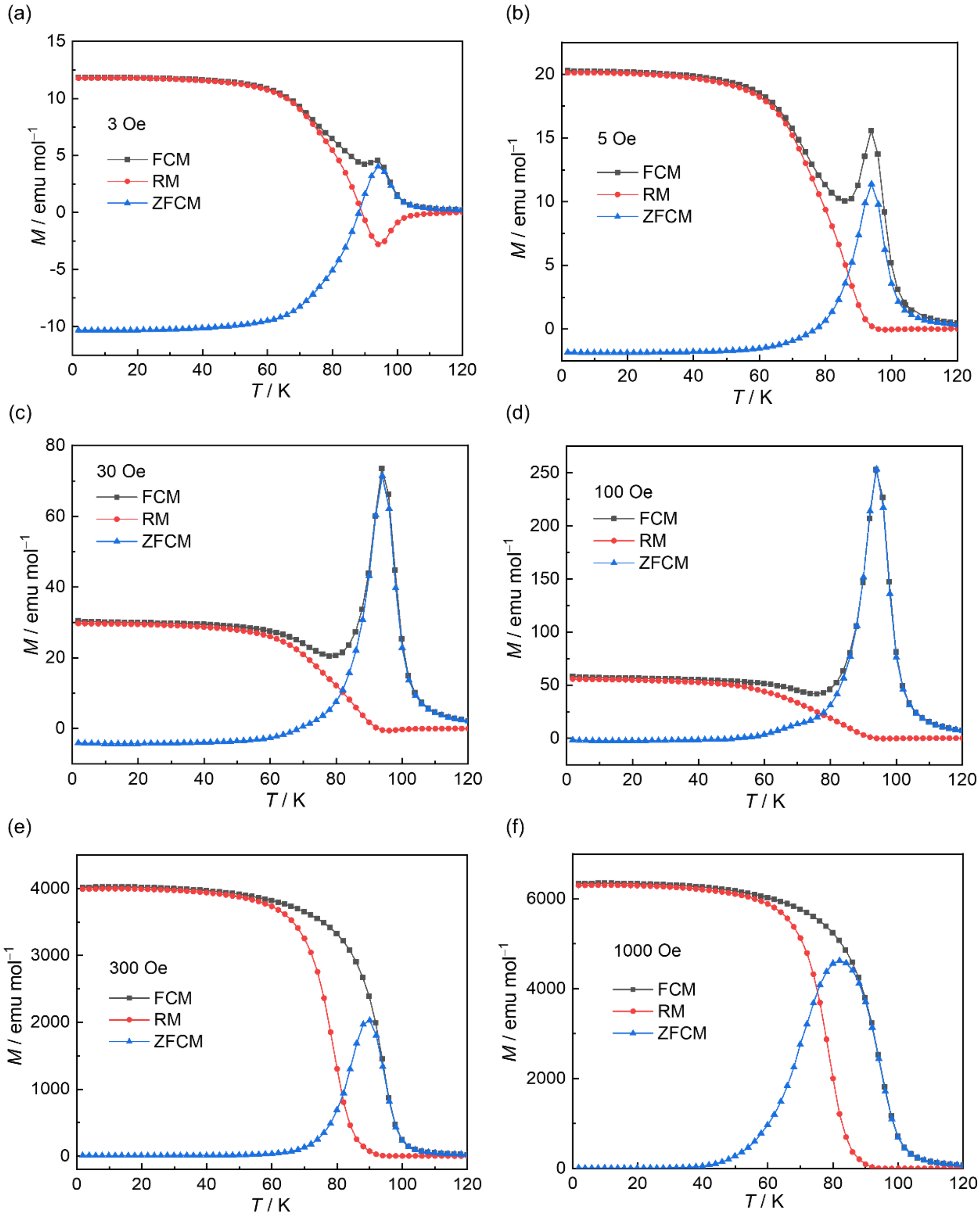

Figure S18. Field-cooled magnetization (FCM), zero-field-cooled magnetization (ZFCM), and remnant magnetization (RM) curves of **1** measured from 120 K to 1.8 K under different dc fields.

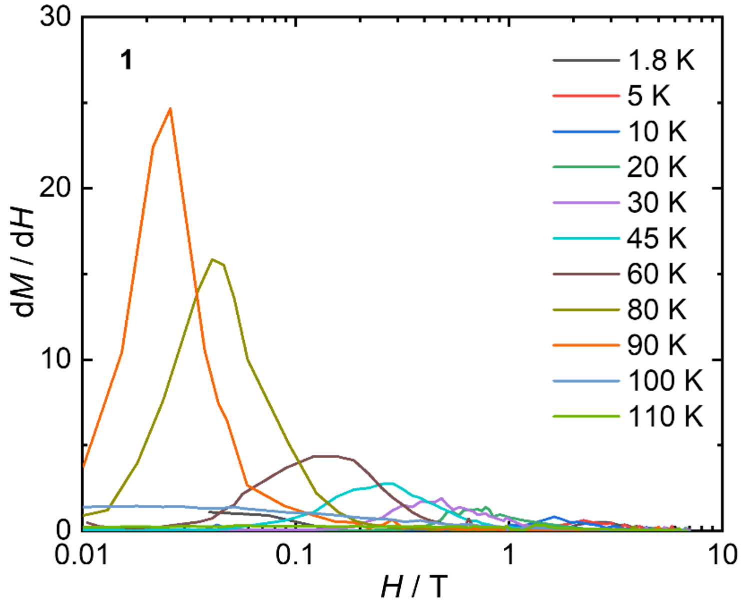

Figure S19. d*M*/d*H* of **1** for the initial field sweep from 0 to 7 T at different temperatures.

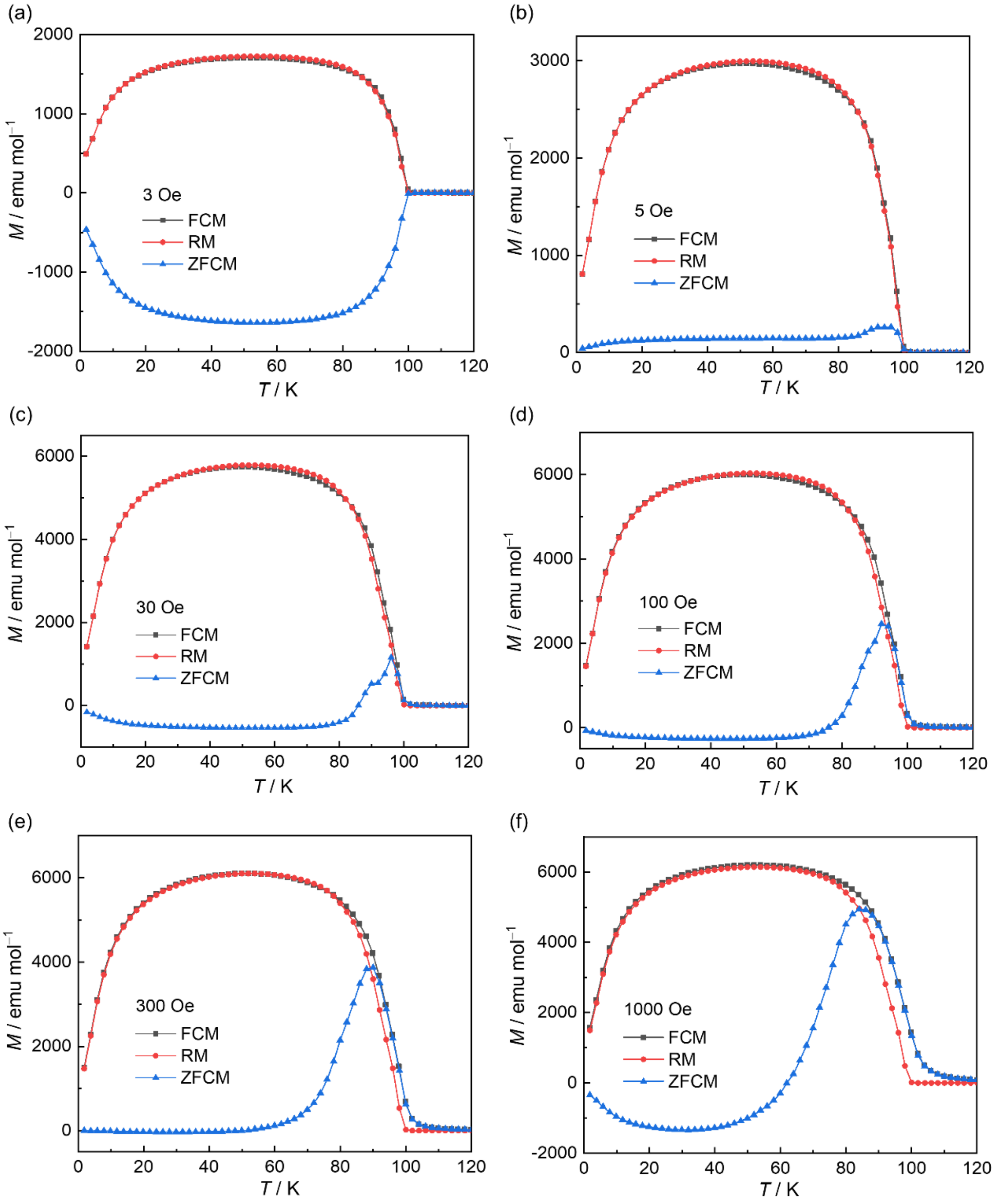

Figure S20. Field-cooled magnetization (FCM), zero-field-cooled magnetization (ZFCM), and remnant magnetization (RM) curves of **2** measured from 120 K to 1.8 K under different dc fields.

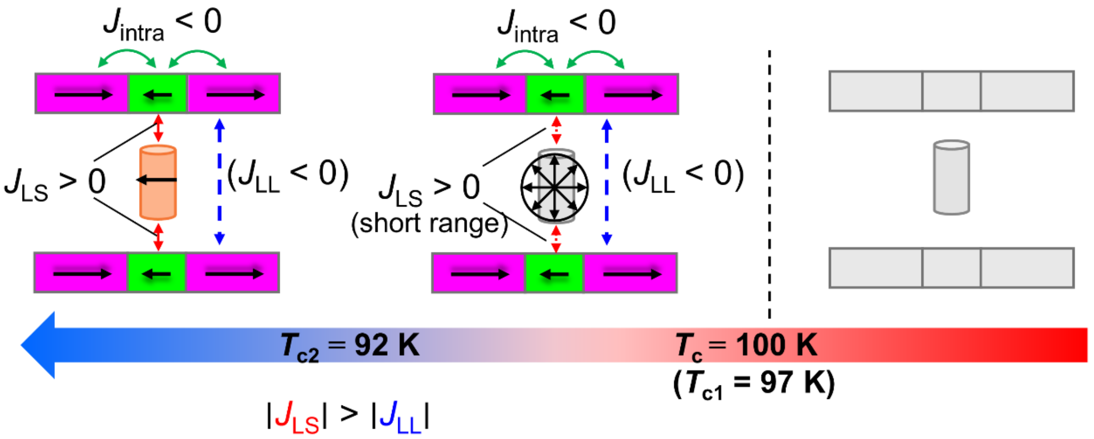

Figure S21. Schematic representation of stepwise spin ordering processes in **2** upon cooling (from right to left), where the purple, green and orange units represent $[Ru_2^{II,II}]$ with S = 1, $TCNQ^{\bullet-}$ with S = 1/2 and $[FeCp^*_2]^+$ with S = 1/2, respectively; gray components mean that units showing paramagnetic behavior.

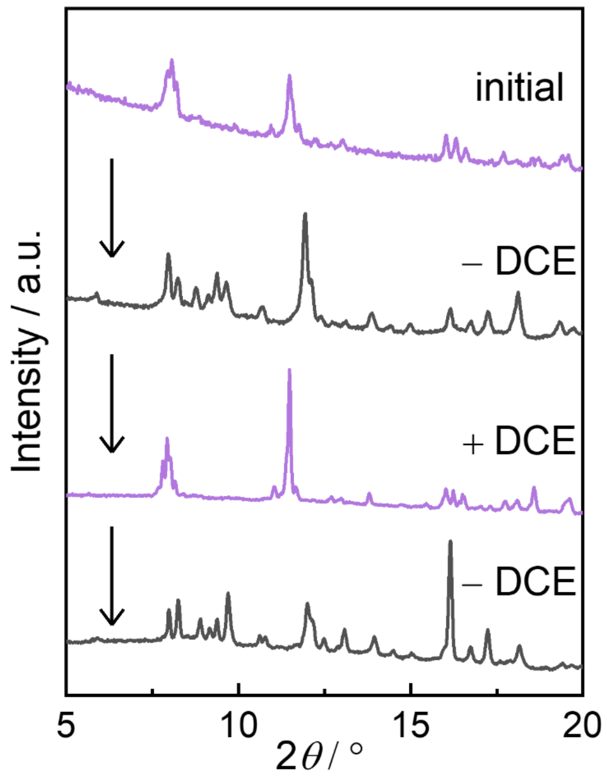

Figure S22. PXRD patterns of the solvated phase (**1-DCE**, purple curves) and the solvent-free phase (**1**, gray curves) in the de-/adsportion cycle of DCE.

References in SI

[1] S. Furukawa, S. Kitagawa, Neutral Paddlewheel Diruthenium Complexes with Tetracarboxylates of Large π-Conjugated Substituents: Facile One-Pot Synthesis, Crystal Structures, and Electrochemical Studies. *Inorg. Chem.* **2004**, *43*, 6464.

[2] F. A. Cotton, R. A. Walton, Multiple Bonds Between Metal Atoms, 2nd ed., Oxford University Press, Oxford, **1993**.

[3] T. J. Kistenmacher, T. J. Emge, A. Bloch, D. Cowan, Structure of the Red, Semiconducting form of 4,4',5,5'-tetramethyl-$\Delta^{2,2'}$-bi-1,3-diselenole-7,7,8,8-Tetracyano-*p*-quinodimethane, TMTSF-TCNQ*. *Acta Crystallographica Section B: Structural Crystallography and Crystal Chemistry* **1982**, *38*, 1193.

[4] R. E. Long, R. A. Sparks, K. N. Trueblood, The Crystal and Molecular Structure of 7,7,8,8-Tetracyanoquinodimethane*. *Acta Crystallographica* **1965**, *18*, 932.

[5] A. Hoekstra, T. Spoelder, A. Vos, The crystal structure of rubidium-7, 7, 8, 8-tetracyanoquinodimethane, Rb-TCNQ, at −160°C. *Acta Crystallographica Section B: Structural Crystallography and Crystal Chemistry* **1972**, *28*, 14.

[6] C. J. Fritchie, P. Arthur, A refinement of the crystal structure of cesium tetracyanoquinodimethanide. *Acta Crystallographica* **1966**, *21*, 139.

[7] V. K. Anand, D. T. Adroja, A. D. Hillier, Ferromagnetic cluster spin-glass behavior in $PrRhSn_3$. *Phys. Rev. B* **2012**, *85*, 014418.

[8] J. Kroder, J. Gooth, W. Schnelle, G. H. Fecher, C. Felser, Observation of spin glass behavior in chiral $Mn_{48}Fe_{34}Si_{18}$ with a β-Mn related structure. *AIP Advances* **2019**, *9*, 055327.

[9] S. Pakhira, C. Mazumdar, R. Ranganathan, S. Giri, M. Avdeev, Large magnetic cooling power involving frustrated antiferromagnetic spin-glass state in R_2NiSi_3 (R = Gd, Er). *Phys. Rev. B* **2016**, *94*, 104414.

[10] H. Fukunaga, W. Kosaka, H. Nemoto, K. Taniguchi, S. Kawaguchi, K. Sugimoto, H. Miyasaka, Magnetic Correlation Engineering in Spin-Sandwiched Layered Magnetic Frameworks. *Chem. Eur. J.* **2020**, *26*, 16755.

[11] W. Kosaka, M. Itoh, H. Miyasaka, Metamagnetism with T_N = 97 K in a layered assembly of paddlewheel [Ru_2] units and TCNQ: an empirical rule for interlayer distances determining the magnetic ground state. *Materials Chemistry Frontiers* **2018**, *2*, 497.

[12] N. Motokawa, S. Matsunaga, S. Takaishi, H. Miyasaka, M. Yamashita, K. R. Dunbar, Reversible Magnetism between an Antiferromagnet and a Ferromagnet Related to Solvation/Desolvation in a Robust Layered $[Ru_2]_2$TCNQ Charge-Transfer System. *J. Am. Chem. Soc.* **2010**, *132*, 11943.

[13] J. Zhang, W. Kosaka, Y. Kitagawa, H. Miyasaka, A Host–Guest Electron Transfer Mechanism for Magnetic and Electronic Modifications in a Redox-Active Metal–Organic Framework. *Angew. Chem. Int. Ed.* **2022**, *61*, e202115976.

[14] Origin(Pro), Version *2018*. OriginLab Corporation, Northampton, MA, USA.
